# Enoxaparin for primary thromboprophylaxis in ambulatory patients with coronavirus disease-2019 (the OVID study): a structured summary of a study protocol for a randomized controlled trial

**DOI:** 10.1186/s13063-020-04678-4

**Published:** 2020-09-09

**Authors:** Stefano Barco, Roland Bingisser, Giuseppe Colucci, André Frenk, Bernhard Gerber, Ulrike Held, Francois Mach, Lucia Mazzolai, Marc Righini, Thomas Rosemann, Tim Sebastian, Rebecca Spescha, Stefan Stortecky, Stephan Windecker, Nils Kucher

**Affiliations:** 1 Clinic of Angiology, University Hospital Zurich, Zurich, Switzerland; 2 Emergency Department, University Hospital Basel, Basel, Switzerland; 3 Service of Haematology, Clinica Luganese Moncucco, Lugano, Switzerland; 4 Department of Cardiology, Inselspital Bern, University of Bern, Bern, Switzerland; 5 Clinic of Haematology, Oncology Institute of Southern Switzerland, Bellinzona, Switzerland; 6 Epidemiology, Biostatistics and Prevention Institute, University of Zurich, Zurich, Switzerland; 7 Cardiology Division, Geneva University Hospital, Geneva, Switzerland; 8 Department of Angiology, Lausanne University Hospital, Lausanne, Switzerland; 9 Division of Angiology and Haemostasis, Department of Medical Specialties, Geneva University Hospital, Geneva, Switzerland; 10 Institute of Primary Care, University of Zurich, Zurich, Switzerland

**Keywords:** COVID-19, Randomised controlled trial, protocol, enoxaparin, anticoagulation, elderly, venous thromboembolism, thrombosis, prevention

## Abstract

**Objectives:**

The OVID study will demonstrate whether prophylactic-dose enoxaparin improves survival and reduces hospitalizations in symptomatic ambulatory patients aged 50 or older diagnosed with COVID-19, a novel viral disease characterized by severe systemic, pulmonary, and vessel inflammation and coagulation activation.

**Trial design:**

The OVID study is conducted as a multicentre open-label superiority randomised controlled trial.

**Participants:**

Inclusion Criteria

1. Signed patient informed consent after being fully informed about the study’s background.

2. Patients aged 50 years or older with a positive test for SARS-CoV2 in the past 5 days and eligible for ambulatory treatment.

3. Presence of respiratory symptoms (i.e. cough, sore throat, or shortness of breath) or body temperature >37.5° C.

4. Ability of the patient to travel to the study centre by private transportation, performed either by an accompanying person from the same household or by the patient themselves

5. Ability to comply with standard hygiene requirements at the time of in-hospital visit, including a face mask and hand disinfectant.

6. Ability to walk from car to study centre or reach it by wheelchair transport with the help of an accompanying person from the same household also complying with standard hygiene requirements.

7. Ability to self-administer prefilled enoxaparin injections after instructions received at the study centre or availability of a person living with the patient to administer enoxaparin.

Exclusion Criteria

1. Any acute or chronic condition posing an indication for anticoagulant treatment, e.g. atrial fibrillation, prior venous thromboembolism (VTE), acute confirmed symptomatic VTE, acute coronary syndrome.

2. Anticoagulant thromboprophylaxis deemed necessary in view of the patient's history, comorbidity or predisposing strong risk factors for thrombosis:

a. Any of the following events occurring in the prior 30 days: fracture of lower limb, hospitalization for heart failure, hip/knee replacement, major trauma, spinal cord injury, stroke,

b. previous VTE,

c. histologically confirmed malignancy, which was diagnosed or treated (surgery, chemotherapy, radiotherapy) in the past 6 months, or recurrent, or metastatic, or inoperable.

3. Any clinically relevant bleeding (defined as bleeding requiring hospitalization, transfusion, surgical intervention, invasive procedures, occurring in a critical anatomical site, or causing disability) within 30 days prior to randomization or sign of acute bleeding.

4. Intracerebral bleeding at any time in the past or signs/symptoms consistent with acute intracranial haemorrhage.

5. Haemoglobin <8 g/dL and platelet count <50 x 10^9^ cells/L confirmed by recent laboratory test (<90 days).

6. Subjects with any known coagulopathy or bleeding diathesis, including known significant liver disease associated with coagulopathy.

7. Severe renal insufficiency (baseline creatinine clearance <30 mL/min calculated using the Cockcroft-Gault formula) confirmed by recent laboratory test (<90 days).

8. Contraindications to enoxaparin therapy, including prior heparin-induced thrombocytopenia and known hypersensitivity.

9. Current use of dual antiplatelet therapy.

10. Participation in other interventional studies over the past 30 days.

11. Non-compliance or inability to adhere to treatment or lack of a family environment or support system for home treatment.

12. Cognitive impairment and/or inability to understand information provided in the study information.

Patient enrolment will take place at seven Swiss centres, including five university hospitals and two large cantonal hospitals.

**Intervention and comparator:**

Patients randomized to the intervention group will receive subcutaneous enoxaparin at the recommended dose of 4,000 IU anti-Xa activity (40 mg/0.4 ml) once daily for 14 days. Patients randomized to the comparator group will receive no anticoagulation.

**Main outcomes:**

Primary outcome: a composite of any hospitalization or all-cause death occurring within 30 days of randomization.

Secondary outcomes: (i) a composite of cardiovascular events, including deep vein thrombosis (including catheter-associated), pulmonary embolism, myocardial infarction/myocarditis, arterial ischemia including mesenteric and extremities, acute splanchnic vein thrombosis, or ischemic stroke within 14 days, 30 days, and 90 days of randomization; (ii) each component of the primary efficacy outcome, within 14 days, 30 days, and 90 days of randomization; (iii) net clinical benefit (accounting for the primary efficacy outcome, composite cardiovascular events, and major bleeding), within 14 days, 30 days, and 90 days of enrolment; (iv) primary efficacy outcome, within 14 days, and 90 days of enrolment; (v) disseminated intravascular coagulation (ISTH criteria, in-hospital diagnosis) within 14 days, 30 days, and 90 days of enrolment.

**Randomisation:**

Patients will undergo block stratified randomization (by age: 50-70 vs. >70 years; and by study centre) with a randomization ratio of 1:1 with block sizes varying between 4 and 8. Randomization will be performed after the signature of the informed consent for participation and the verification of the eligibility criteria using the electronic data capture software (REDCAP, Vanderbilt University, v9.1.24).

**Blinding (masking):**

In this open-label study, no blinding procedures will be used.

**Numbers to be randomised (sample size):**

The sample size calculation is based on the parameters *α* = 0.05 (2-sided), power: 1−*β* = 0.8, event rate in experimental group, *pexp* = 0.09 and event rate in control group, *pcon* = 0.15. The resulting total sample size is 920. To account for potential dropouts, the total sample size was fixed to 1000 with 500 patients in the intervention group and 500 in the control group.

**Trial Status:**

Protocol version 1.0, 14 April 2020. Protocol version 3.0, 18 May 2020

Recruiting start date: June 2020.

Last Patient Last Visit: March 2021.

**Trial registration:**

ClinicalTrials.gov Identifier: NCT04400799

First Posted: May 26, 2020

Last Update Posted: July 16, 2020

**Full protocol:**

The full protocol is attached as an additional file, accessible from the Trials website (Additional file 1). In the interest in expediting dissemination of this material, the familiar formatting has been eliminated; this Letter serves as a summary of the key elements of the full protocol.

## Supplementary information

**Additional file 1.**

## Data Availability

All the Steering Committee members and co-authors will have access to the original dataset. The data will be available from the author on reasonable request (nils.kucher@usz.ch).

