# Supplementary material for: Enoxaparin for primary thromboprophylaxis in ambulatory patients with coronavirus disease-2019 (the OVID study): a structured summary of a study protocol for a randomized controlled trial
Source: Trials. 2020 Sep 9;21:770. doi: 10.1186/s13063-020-04678-4 (PMC7479300; doi:10.1186/s13063-020-04678-4)
Supplement: Supplementary file 1 — Additional file 1. [file 13063_2020_4678_MOESM1_ESM.pdf]

Clinical Study Protocol

ENOXAPARIN FOR PRIMARY THROMBOPROPHYLAXIS IN AMBULATORY PATIENTS WITH CORONAVIRUS: THE MULTICENTER RANDOMIZED CONTROLLED *OVID* TRIAL

**Study Type:**	Intervention with Investigational Medicinal Product (IMP)
**Study Categorisation:**	Clinical Trial with IMP Category B
**Study Registration:**	To be defined
**Study Identifier:**	OVID trial
**Sponsor-Investigator:**	University of Zurich<br>Prof. Dr. med. Nils Kucher<br>University Hospital Zürich<br>Clinic for Angiology<br>Rämistrasse 100<br>8091 Zürich (Switzerland)
**Investigational Product:**	Enoxaparin (Clexane®)
**Protocol Version and Date:**	3.0 / 18.05.2020

CONFIDENTIAL

The information contained in this document is confidential and the property of Clinic of Angiology at the University Hospital Zurich. The information may not - in full or in part - be transmitted, reproduced, published, or disclosed to others than the applicable Independent Ethics Committee(s) and Competent Authority(ies) without prior written authorization from Clinic of Angiology at the University Hospital Zurich, except to the extent necessary to obtain informed consent from those participants who will participate in the study.

SIGNATURE PAGE

**Study number**	To be defined
**Study Title**	ENOXAPARIN FOR PRIMARY THROMBOPROPHYLAXIS IN AMBULATORY PATIENTS WITH CORONAVIRUS: THE MULTICENTER RANDOMIZED CONTROLLED *OVID* TRIAL

The Sponsor-Investigator and trial statistician have approved the protocol version 3.0 - 18.05.2020, and confirm hereby to conduct the study according to the protocol, current version of the World Medical Association Declaration of Helsinki, ICH-GCP guidelines and the local legally applicable requirements.

Sponsor-Investigator:

Prof. Dr. med Nils Kucher
University Zurich
University Hospital Zurich
Clinic of Angiology
Rämistrasse 100
CH 8091 Zürich, Switzerland
Phone: +41 44 255 4082
E-Mail: nils.kucher@usz.ch

Zürich, 19.5.2020

Place/Date Signature

Biometrician:

PD Dr. Ulrike Held
University of Zurich
Dept. of Biostatistics
Epidemiology, Biostatistics and Prevention Institute
Hirschengraben 84
8001 Zurich, Switzerland

Phone +41 44 634 45 39
E-Mail: ulrike.held@uzh.ch

Zürich, 19.5.2020

Place/Date Signature

Local Principal Investigator at study site:

I have read and understood this trial protocol and agree to conduct the trial as set out in this study protocol, the current version of the World Medical Association Declaration of Helsinki, ICH-GCP guidelines and the local legally applicable requirements.

Site

Prof. Dr. med. Nils Kucher
University Hospital Zürich
Clinic of Angiology
Rämistrasse 100
8091 Zürich (Switzerland)

Principal investigator Nils Kucher

Zürich, 19.5.2020

Place/Date *Signature*

Coordinating investigator

Dr. sc. nat. Rebecca Spescha
University Hospital Zürich
Clinic of Angiology
Rämistrasse 100
8091 Zürich (Switzerland)
E-Mail: Rebecca.spescha@usz.ch

Zürich, 19.05.2020

Place/Date *Signature*

Local Principal Investigator at study site:

I have read and understood this trial protocol and agree to conduct the trial as set out in this study protocol, the current version of the World Medical Association Declaration of Helsinki, ICH-GCP guidelines and the local legally applicable requirements.

**Site**	External Sites
**Principal investigator**	First name Name

_______________________ _______________________________

Place/Date *Signature*

TABLE OF CONTENTS

SIGNATURE PAGE 2
STUDY SYNOPSIS 8
LIST OF ABBREVIATIONS AND DEFINITIONS OF TERMS 15
STUDY SCHEDULE 16
1 STUDY ADMINISTRATIVE STRUCTURE 18
1.1 Sponsor-Investigator 18
1.2 Investigators 18
1.3 Principal Investigator(s)/ Study Sites 19
1.4 Statistician 19
1.5 Monitoring Institution 19
1.6 Data Safety Monitor Board 20
2 ETHICS AND REGULATORY ASPECTS 20
2.1 Study Registration 20
2.2 Categorisation of Study 20
2.3 Competent Ethics Committee (CEC) 20
2.4 Competent Authorities (CA) 21
2.5 Ethical Conduct of the Study 21
2.6 Declaration of Interest 21
2.7 Participant Information and Informed Consent 21
2.8 Participant Privacy and Confidentiality 22
2.9 Early Termination of the Study 22
2.10 Protocol Amendments 22
3 INTRODUCTION 23
3.1 Background and Rationale 24
3.2 Investigational Product and Indication 30
3.3 Preclinical Evidence 31
3.4 Clinical Evidence to Date and post-market experience 32
3.5 Dose Rationale 32
3.6 Explanation for Choice of Comparator 32
3.7 Risk/benefits 32
3.8 Study population 34
4 STUDY OBJECTIVES 35
4.1 Overall Objective 35
4.2 Primary Objective 35
4.3 Secondary Objectives 35
4.4 Safety Objectives 35
5 STUDY OUTCOMES 36
5.1 Primary Outcome 36
5.2 Secondary Outcomes 36
5.3 Other Outcomes of Interest 36

5.4 Safety Outcomes....36
6 STUDY DESIGN AND COURSE OF STUDY....37
6.1 General Study Design and Justification of the Design....37
6.2 Study Duration and Study Schedule....37
6.3 Methods of Minimising Bias....37
6.3.1 Randomisation....37
6.3.2 Blinding Procedures....37
6.3.3 Other Methods of Minimising Bias....38
6.4 Unblinding Procedures (Code break)....38
7 STUDY POPULATION....38
7.1 Eligibility Criteria....38
7.1.1 Inclusion Criteria....38
7.1.2 Exclusion Criteria....38
7.2 Recruitment and Screening....39
7.3 Assignment to Study Groups....43
7.4 Criteria for Withdrawal/ Discontinuation of Participants....43
8 STUDY INTERVENTION....43
8.1 Identity of Investigational Product(s)....43
8.1.1 Experimental Intervention....43
8.1.2 Control Intervention....46
8.1.3 Packaging, Labelling and Supply (Re-Supply)....46
8.1.4 Storage Conditions....47
8.2 Administration of experimental and control interventions....47
8.2.1 Experimental Intervention....47
8.2.2 Control Intervention....47
8.3 *Dose Modifications*....47
8.4 Compliance with Study Intervention....48
8.5 Data Collection and Follow-up for Withdrawn Participants....48
8.6 Trial Specific Preventive Measures....48
8.7 Concomitant Interventions....48
8.8 Study Drug Accountability....49
8.9 Return or Destruction of Study Drug....49
9 STUDY ASSESSMENTS....49
9.1 Study Flow Chart/Table of Study Procedures and Assessments....49
9.2 Assessments of Outcomes....50
9.2.1 Assessment of Primary Outcome....50
9.2.2 Assessment of Secondary Outcomes....50
9.2.3 Assessment of Safety Outcomes....50
9.2.4 Assessments in Participants Who Prematurely Stop the Study....51
9.3 Procedures at each visit....51
9.3.1 Pre-screening period (Day -5 to Day 1)....51
9.3.2 Screening and baseline Visit (Day 1)....51
9.3.3 Follow-up visits, including Final visit....52
10 SAFETY....52

10.1 Definitions 52
10.2 Recording of Serious Adverse Events 53
10.3 Assessment of Serious Adverse Events 53
10.4 Reporting of Serious Adverse Events 54
10.1 Data Safety Monitoring Board (DSMB) 55
10.2 Follow up of Serious Adverse Events 55
11 STATISTICAL METHODS 56
11.1 Hypothesis 56
11.2 Determination of Sample Size 56
11.3 Statistical Criteria of Termination of Trial 57
11.4 Planned Analyses 57
11.4.1 Datasets to be Analysed, Analysis Populations......57
11.4.2 Primary Analysis 57
11.4.3 Secondary Analyses 57
11.4.4 Interim Analyses 58
11.4.5 Safety Analysis 59
12 ELIGIBILITY OF THE PROJECT SITE(S) 59
13 DATA QUALITY ASSURANCE AND CONTROL 59
13.1 DATA HANDLING AND RECORD KEEPING 60
13.1.1 Case Report Forms 60
13.1.2 Specification of Source Documents 60
13.1.3 Record Keeping / Archiving......61
13.2 Data Management 61
13.2.1 Data Management System......61
13.2.2 Data Security, Access and Back-up 62
13.2.3 Analysis and Archiving......62
13.2.4 Electronic and Central Data Validation......62
13.3 Monitoring 63
13.4 Audits and Inspections 63
13.5 Confidentiality, Data Protection 63
13.6 Storage of Biological Material and Related Health Data 63
14 PUBLICATION AND DISSEMINATION POLICY 63
15 FUNDING AND SUPPORT 64
15.1 Funding 64
15.2 Other Support 64
16 INSURANCE 64
17 REFERENCES 64

STUDY SYNOPSIS

**Sponsor / Sponsor-Investigator**	University of Zurich<br>Prof. Dr. med. Nils Kucher<br>University Hospital Zürich<br>Clinic for Angiology<br>Rämistrasse 100
**Study Title:**	ENOXAPARIN FOR PRIMARY THROMBOPROPHYLAXIS IN AMBULATORY PATIENTS WITH CORONAVIRUS: THE MULTICENTER RANDOMIZED CONTROLLED OVID TRIAL
**Short Title / Study ID:**	OVID trial
**Protocol Version and Date:**	3.0 / 18.05.2020
**Trial registration:**	Pending
**Study category and Rationale**	Clinical study with IMP Category B.
**Clinical Phase:**	Clinical Phase IIIb.

**Background and Rationale:**	Coronavirus disease (COVID-19) has emerged as a pandemic and a public health crisis of global proportions. As of April 9, 2020, a total of 1,716,371 COVID-19 cases have been diagnosed with the severe acute respiratory syndrome corona virus 2 (SARS-CoV-2) across more than 170 countries. Of 1,223,520 active cases, 1,173,564 are in a mild and 49,956 are in a serious or critical condition. Of 492,851 closed cases, 103,847 have died and 389,004 recovered or were discharged from hospital (Source: WHO Daily Briefing on COVID-19; via www.worldometers.info/coronavirus).<br><br>One of the most frequently described poor prognostic features in patients with COVID-19 is the development of coagulopathy. An increase in D-dimers, which reflects the presence of disseminated intravascular coagulation, is usually observed in the most severe cases of COVID-19 and it correlates with in-hospital mortality (2-4). In hospitalized patients with sepsis-induced coagulopathy or with markedly elevated D-dimer associated with COVID-19 infection, anticoagulation with low-molecular-weight heparin (LMWH) was associated with lower mortality.<br><br>COVID-19 emerged as a dramatic prothrombotic condition and it appears now clear, a few months after the first patients described in China, that COVID-19 patients are characterized by a substantial risk of developing acute pulmonary embolism (PE) or deep vein thrombosis (DVT) due to local and systemic inflammation, reduced mobility, hypoxia, endothelial dysfunction.<br>In particular, the cumulative incidence of VTE in COVID-19 patients is approximately 20-30% (up to 70% in ICU patients undergoing VTE screening). This contrasts with the risk of VTE observed in medically ill patients (<3%).<br>Moreover, half of the VTE events, mostly PE, were diagnosed at hospital admission, suggesting that these events developed during home quarantine.<br><br>Recent guidances stated that prophylactic-dose LMWH, such as enoxaparin, should be considered in all patients who require hospital admission for COVID-19 in the absence of any contraindications. One of the better known non-anticoagulant properties of heparins, their anti-inflammatory function, include binding to inflammatory cytokines, inhibition of neutrophil chemotaxis and leukocyte migration, neutralization of the positively charged peptide complement factor C5a, and sequestering acute phase proteins: this may provide a benefit in COVID infection where proinflammatory cytokines are markedly raised and acute respiratory distress syndrome represents a feared and life-threatening complication.<br><br>COVID-19 patients are characterized by systemic inflammation, reduced mobility, hypoxia, endothelial dysfunction, signs of myocarditis and heart failure, and the underlying coagulopathy, all factors increasing the risk of developing thromboembolic events.<br><br>It remains unclear whether COVID-19 patients not admitted to the hospital due to non-severe clinical conditions should receive thromboprophylaxis and whether this provides a clinical benefit

	weighed against the risk of anticoagulant-associated bleeding. The evidence is scarce also for non-COVID-19 patients. The most recent American Society of Hematology (ASH) guidelines state that "In medical outpatients with minor provoking risk factors for VTE (eg, immobility, minor injury, illness, infection), the ASH guideline panel suggests not using VTE prophylaxis (conditional recommendation, very low certainty in the evidence of effect)."<br><br>Our hypothesis is that enoxaparin may prevent or limit coagulopathy, including the occurrence of thromboembolic events, in the presence of a mild COVID disease in an outpatient setting.
**Objective(s):**	The OVID study will show whether prophylactic-dose enoxaparin improves survival and reduces any hospitalizations in ambulatory patients aged 50 or older diagnosed with COVID-19, a novel viral disease characterized by severe systemic, pulmonary, and vessel inflammation and coagulation activation.
**Primary Outcome:**	The primary efficacy outcome is a composite of any hospitalization or all-cause death occurring within 30 days of randomization.
**Study design:**	The study will be conducted as a multicentre randomized open-label controlled trial. In the study, a total of 1,000 adult patients aged 50 or older with COVID-19 and candidates to ambulatory treatment will be randomized to receive enoxaparin 40 mg sc qD or no treatment for a total of 14 days. The primary outcome will be assessed within 30 days of enrolment.

**Inclusion / Exclusion criteria:**	*Inclusion Criteria*<br>1) Signs patient informed consent after being fully informed about the study's background.<br>2) Patients aged 50 years or older with a positive test for SARS-CoV2 in the past 5 days and eligible for ambulatory treatment.<br>3) Presence of respiratory symptoms (i.e. cough, sore throat, or shortness of breath) or body temperature >37.5° C.<br>4) Ability of the patient to travel to the study center by private transportation, performed either by accompanying person from same household or by the patient him/herself.<br>5) Ability to comply with standard hygiene requirements at the time of in-hospital visit, including a face mask and hand disinfectant.<br>6) Ability to walk from car to study center or reach it by wheel chair transport with the help of an accompanying person from the same household also complying with standard hygiene requirements.<br>7) Ability to self-administer prefilled enoxaparin injections after instructions received at the study center or availability of a person living with the patient to administer enoxaparin.<br><br>*Exclusion Criteria*<br>1) Any acute or chronic condition posing an indication for anticoagulant treatment, e.g. atrial fibrillation, prior VTE, acute confirmed symptomatic VTE, acute coronary syndrome.<br>2) Anticoagulant thromboprophylaxis deemed necessary in view of the patient's history, comorbidity or predisposing strong risk factors for thrombosis:<br>a. Any of the following events occurring in the prior 30 days: fracture of lower limb, hospitalization for heart failure, hip/knee replacement, major trauma, spinal cord injury, stroke,<br>b. previous VTE,<br>c. histologically confirmed malignancy, which was diagnosed or treated (surgery, chemotherapy, radiotherapy) in the past 6 months, or recurrent, or metastatic, or inoperable.<br>3) Any clinically relevant bleeding (defined as bleeding requiring hospitalization, transfusion, surgical intervention, invasive procedures, occurring in a critical anatomical site, or causing disability) within 30 days prior to randomization or sign of acute bleeding.<br>4) Intracerebral bleeding at any time in the past or signs/symptoms consistent with acute intracranial hemorrhage.<br>5) Hemoglobin <8 g/dL and platelet count <50 x 10^9 cells/L confirmed by recent laboratory test (<90 days).<br>6) Subjects with any known coagulopathy or bleeding diathesis, including known significant liver disease associated with coagulopathy.<br>7) Severe renal insufficiency (baseline creatinine clearance <30 mL/min calculated using the Cockcroft-Gault formula) confirmed by recent laboratory test (<90 days).<br>8) Contraindications to enoxaparin therapy, including prior heparin-induced thrombocytopenia and known hypersensitivity.<br>9) Current use of dual antiplatelet therapy.<br>10) Participation in other interventional studies over the past 30 days.<br>11) Non-compliance or inability to adhere to treatment or lack of

	a family environment or support system for home treatment.<br>12) Cognitive impairment and/or inability to understand to information provided in the study information.
**Measurements and procedures:**	We implemented two logistical solutions to integrate the process of SARS-CoV2 testing, pre-screening, screening (hot-line and flyers), in-hospital recruitment, enrolment and randomization/allocation. A nationwide OVID Hot-Line telephone number will be made available in 3 languages (German, French, Italian) for interested patients or test centers to contact the Hot-Line. Standard hygiene precautions will be met at the study centers to avoid spreading of SARS-CoV2 among other patients or health care workers. Principles of patient and investigator safety will be applied. Standard procedures concerning privacy, discussion with patients on details of the study, collection of informed consent, and instruction on how to administer the study medication will be maintained in conformity with GCP recommendations. This will also include outcome measurements to be conducted by telephone with standardized questionnaire.
**Study Product / Intervention:**	Enoxaparin (Clexane®) will be given at the recommended dose of 4,000 IU antiXa activity (40 mg/0.4 ml) once daily by SC injection for 14 days.
**Control Intervention (if applicable):**	No study drug.

**Number of Participants with Rationale:**	The sample size calculation is based on the parameters α= 0.05 (2-sided), power = $1-\beta$ = 0.8, event rate in experimental group, $pexp$= 0.09 and event rate in control group, $pcon$= 0.15. The resulting total sample size is 920. To account for potential drop-outs, the total sample size was fixed to 1000. This will be the maximum sample size, no increase in sample size is planned. Results will be reported in terms of risk ratios (RR) between experimental and control group, i.e. we anticipate that the estimated RR will be < 1.<br><br>We obtained official data on fatality and hospitalization rates observed in the Swiss population until 31.03.2020: a total of 12.372 patients aged 50 years or older tested positive for SARS-CoV-2, of whom 645 (5.2%) died and 2.350 (19.0%) were hospitalized irrespective of whether this consisted of a primary hospitalization (after evaluation at the emergency department) or a secondary hospitalization for clinical deterioration after initial ambulatory treatment. Assuming that two thirds of deaths and of any hospitalization occurred in ambulatory patients, we estimated that the primary efficacy outcome rate would occur in 15% (any hospitalization 11%, case fatality rate 4%). As we anticipate that a substantial number of the primary endpoint is due to venous or arterial thromboembolic complications, which would be prevented by the use of prophylactic-dose enoxaparin, we estimated that enoxaparin will decrease the primary efficacy outcome to 9% (RR 0.6).<br>Prior studies of thromboprophylaxis in hospitalized patients with medical illnesses were also considered for estimating the benefit of enoxaparin use in medical patients and sample size calculation.
**Study Duration:**	10 months
**Study Schedule:**	1. Submission to HA/EC 06/05/2020<br>2. First Patient, First Visit date: 25/05/2020<br>3. First Patient Last Visit date: 14/10/2020<br>4. Last Patient Last Visit date: 14/03/2021<br>5. Report(s) date 14/04/2021<br>6. Planned Publication(s) date 14/05/2021
**Investigator(s):**	Prof. Dr. med Nils Kucher<br>University Zurich<br>University Hospital Zurich<br>Clinic of Angiology<br>Rämistrasse 100<br>CH 8091 Zürich, Switzerland<br><br>Phone: +41 44 255 4082<br>E-Mail: nils.kucher@usz.ch
**Study Centre(s):**	All five Swiss university hospitals and two large cantonal hospitals.

**Statistical Considerations:**	The primary efficacy outcome analysis will be conducted in the intention-to-treat (ITT) population, consisting of all randomized subjects who signed a valid informed consent. Descriptive statistics of the patient characteristics at baseline will include mean and standard deviation for continuous variables, median and interquartile range for the ordinal or non-normal variables, as well as numbers and percentages of total for the categorical variables. For the primary outcome, the relative risk will be calculated for the experimental group as compared to control group, with 95% confidence interval. Refined analyses include the stratification variables in order to reduce unexplained heterogeneity. For that, the Mantel Haenszel method as well as multiple logistic regression models will be used.<br>A single interim analysis is planned. The aim of the interim analysis is to stop the trial early for efficacy (superiority) or futility.
**GCP Statement:**	This study will be conducted in compliance with the protocol, the current version of the Declaration of Helsinki, the ICH-GCP Guideline as well as all national legal and regulatory requirements.

LIST OF ABBREVIATIONS AND DEFINITIONS OF TERMS

AE	Adverse Event
CA	Competent Authority (e.g. Swissmedic)
CEC	Competent Ethics Committee
ClinO	Clinical Trials Ordinance
eCRF	Electronic Case Report Form
CTCAE	Common terminology criteria for adverse events
DVT	Deep Vein Thrombosis
DSUR	Development safety update report
GCP	Good Clinical Practice
H0	Null hypothesis
H1	Alternative hypothesis
ICH	International Council on Harmonization
ICU	Intensive Care Unit
IMP	Investigational Medicinal Product
ISF	Investigator Site File
ISTH-SSC	
ITT	Intention to Treat
LMWH	low-molecular-weight heparin
PI	Principal Investigator
PE	Pulmonary Embolism
SAE	Serious Adverse Event
SARS	Severe Acute Respiratory Syndrome
SDV	Source Data Verification
SmPC	Summary of Product Characteristics
SNCTP	Swiss National Clinical Trial Portal
SOP	Standard Operating Procedure
SUSAR	Suspected Unexpected Serious Adverse Reaction
TMF	Trial Master File

STUDY SCHEDULE

Study Periods	***Pre-screening (Hotline or site)***	***Screening (Site)***	***Treatment Period***				***Follow-up***	
Visit			*1*	*2*	*3*	*4*	*5*	*6*
Time	Day -5 to Day 1	Day -5 to Day 1	Day 1 (Baseline)	Day 3 ± 1	Day 7 ± 2	Day 14 ± 2	Day 30 ± 3	Day 90 ± 3
PIC1[a]	(x)							
Pre-screen survey	x	x						
Patient Information and Informed Consent (PIC2)			*x*					
Demographics and baseline characteristics[b]			*x*					
Current medications			*x*					
In- / Exclusion Criteria			x					
Vital Signs[c]			x					
Laboratory Tests[d]			*x*					
Randomization			x					
Dispense of Study Medication[e]			*x*					
Primary outcome measures[f]			*x*	*x*	*x*	*x*	*x*	
Secondary outcome measures[g]			*x*	*x*	*x*	*x*	*x*	*x*
Serious adverse events			*x*	*x*	*x*	*x*	*x*	*x*

a. Patient informed consent form 1 (PIC1) is not necessary if patient contacts OVID Hotline or study center directly. It has to be signed if physician contacts Hotline or study center on behalf of the patient.

b. Demographics, medical history.
c. Vital signs (respiratory rate, heart rate, arterial blood pressure, body temperature, arterial oxygen saturation), height, weight.
d. Laboratory tests (blood cell count, hemoglobin and creatinine).
e. The dispense of study medication and the administration of the first dose will be done at the study center after appropriate instruction and under medical supervision. The following doses will be administered from an instructed person of the same household or self-administered at home until Day 14. Instructions on how to administer enoxaparin will also made be available in paper and video from the product manufacturer.
f. Any hospitalization or all-cause death.
g. Pulmonary embolism, myocardial infarction/myocarditis, arterial ischemia including mesenteric and extremities, acute splanchnic vein thrombosis, or ischemic stroke; major bleeding, non-major clinically relevant bleeding.

1 STUDY ADMINISTRATIVE STRUCTURE

1.1 Sponsor-Investigator

Prof. Dr. med Nils Kucher
University Zurich
University Hospital Zurich
Clinic of Angiology
Rämistrasse 100
CH 8091 Zürich
Switzerland
Phone: +41 44 255 4082
Fax: +41 44 255 45 10
E-Mail: nils.kucher@usz.chnils

1.2 Investigators

Prof. Stephan Windecker, Head of Cardiology, Inselspital
stephan.windecker@insel.ch

Prof. Francois Mach, Head of Cardiology, HUG
francois.mach@hcuge.ch

Prof. Marc Righini, Head of Angiology, HUG
marc.righini@hcuge.ch

Prof. Lucia Mazzolai, Head of Angiology, CHUV
Lucia.mazzolai@chuv.ch

Prof. Roland Bingisser, Head of Emergency Department, USB
Roland.bingisser@usb.ch

Prof. Nils Kucher, Head of Angiology, USZ
Nils.kucher@usz.ch

Dr. Stefano Barco, PhD, Angiology, USZ
Stefano.barco@usz.ch

Andre Frenk, PhD, Cardiology, Inselspital
andre.frenk@insel.ch

Stefan Stortecky, PhD, Invasive Cardiology, Inselspital
stefan.stortecky@insel.ch

PD Bernhard Gerber, MD, Clinic of Hematology, Oncology Institute of Southern Switzerland, Bellinzona
Bernhard.Gerber@eoc.ch

PD Giuseppe Colucci, MD, Clinic of Hematology, Clinica Luganese Moncucco, Lugano
Giuseppe.colucci@moncucco.ch

OVID Logistics support for centers:
Daniela Nosetti, Clinic Manager Angiology USZ
Daniela.nosetti@usz.ch

OVID Swiss public awareness strategy
Cindy Maeder, Head UKOM USZ
Cindy.maeder@usz.ch

1.3 Principal Investigator(s)/ Study Sites

Prof. Dr. med Nils Kucher
University Zurich
University Hospital Zurich
Clinic of Angiology
Rämistrasse 100
CH 8091 Zürich
Switzerland
Phone: +41 44 255 4082
Fax: +41 44 255 45 10
E-Mail: nils.kucher@usz.ch

1.4 Statistician

PD Dr. rer. nat. Ulrike Held
Dept. of Biostatistics - Epidemiology, Biostatistics and Prevention Institute
University of Zurich
Hirschengraben 84
8001 Zurich
Switzerland
Phone +41 44 634 45 39
E-Mail: ulrike.held@uzh.ch

1.5 Monitoring Institution

Clinical Trials Center - Monitoring
Universitätsspital Zürich
Rämistrasse 100 / MOU2
8091 Zürich

1.6 Data Safety Monitor Board

PD Dr. Marc Schindewolf (Angiologie, Inselspital, Bern)
PD Dr. Jacqueline Pichler (Pneumologie, Inselspital, Bern)
Dr. Arnaud Künzi, Statistiker, CTU (Bern)

2 ETHICS AND REGULATORY ASPECTS

Before this study will be conducted, the protocol, the proposed participant information and consent form as well as other study-specific documents will be submitted to a properly constituted Competent Ethics Committee (CEC) and Competent Authorities (Swissmedic) in agreement with local legal requirements, for formal approval. Any amendment to the protocol must as well be approved.

The decision of the CEC and Swissmedic concerning the conduct of the study will be made in writing to the Sponsor-Investigator before commencement of this study. The clinical study can only begin once approval from all required authorities has been received. Any additional requirements imposed by the authorities shall be implemented.

2.1 Study Registration

The study will be registered in the Swiss Federal Complementary Database (Swiss National Clinical Trial Portal (SNCTP) and in the international trial registry ClinicalTrials.gov (clinicaltrials.gov).

2.2 Categorisation of Study

Category B.
The current label of enoxaparin (study medication) does not cover ambulatory patients with acute infectious diseases without additional venous thromboembolism (VTE) risk factors. The efficacy and safety of prophylactic-dose enoxaparin has not been studied in COVID-19 ambulatory patients, as SARS-CoV2 has been described first in 2019.

2.3 Competent Ethics Committee (CEC)

Approval from the appropriate constituted Competent Ethics Committee is sought for each study site in the clinical trial. The reporting duties and allowed time frame are respected. No substantial amendments are made to the protocol without prior Sponsor and CEC approval, except where necessary to eliminate apparent immediate hazards to study participants.
Premature study end or interruption of the study is reported within 15 days. The regular end of the study is reported to the CEC within 90 days, the final study report shall be submitted within one year after study end. Amendments are reported according to chapter 2.10.

2.4 Competent Authorities (CA)

The Sponsor will obtain approval from Swissmedic before the start of the clinical trial. Reporting will be done within the allowed time frame. Planned or premature study end are reported within 90 and 15 days, respectively. The final report will be submitted to the CA within one year after the end of the study. Amendments are reported according to chapter 2.10.

2.5 Ethical Conduct of the Study

The study will be carried out in accordance with principles enunciated in the current version of the Declaration of Helsinki, the guidelines of Good Clinical Practice (GCP) issued by ICH, and Swiss competent authority's requirements.

CEC and competent authorities will receive annual safety and interim reports and be informed about non-substantial amendments, the course of the study, and the study stop/ end in agreement with local requirements.

2.6 Declaration of Interest

No conflicts of interest.

2.7 Participant Information and Informed Consent

The investigator must explain to each participant the nature of the study, its purpose, the procedures involved, the expected duration, the potential risks and benefits and any discomfort it may entail. Each participant must be informed that the participation in the study is voluntary and that he/she may withdraw from the study at any time and that withdrawal of consent will not affect his/her subsequent medical treatment.

The participant must be informed that his/her medical records may be examined by authorized individuals other than their treating physician.

All participants for this study will be provided a participant information sheet and a consent form describing this study and providing sufficient information for participants to make an informed decision about their participation in this study.

The participant information sheet and the consent form will be submitted with the protocol for review and approval for the study by the CEC and by Swissmedic. The formal consent of a participant, using the approved consent form, must be obtained before that participant is submitted to any study procedure.

The participant should read and consider the statement before signing and dating the informed consent form, and should be given a copy of the signed document. The consent form must also be signed and dated by the investigator (or his designee) and it will be retained as part of the study records.

2.8 Participant Privacy and Confidentiality

The investigator affirms and upholds the principle of the participant's right to privacy and that they shall comply with applicable privacy laws. Especially, anonymity of the participants shall be guaranteed when presenting the data at scientific meetings or publishing them in scientific journals.

Individual subject medical information obtained as a result of this study is considered confidential and disclosure to third parties is prohibited. Subject confidentiality will be further ensured by utilising subject identification code numbers to correspond to treatment data in the computer files.

Such medical information may be given to the participant's personal physician or to other appropriate medical personnel responsible for the participant's welfare, if the patient has given his/her written consent to do so.

For data verification purposes, authorised representatives of the Sponsor-Investigator, a competent authority (e.g. Swissmedic), or an ethics committee may require direct access to parts of the medical records relevant to the study, including participants' medical history.

2.9 Early Termination of the Study

The Sponsor-Investigator may terminate the study prematurely according to certain circumstances, e.g.:

- early evidence of benefit or harm of the experimental intervention, e.g. for superiority or futility as defined in Statistical Methods, (Section 10.3);
- insufficient participant recruitment, e.g. <30% of the expected number of patients six months after the enrolment of the first patient, also based on the course of SARS-CoV2 infections in Switzerland;
- when the safety of the participants is doubtful or at risk, respectively, based on recommendations received from DSMB committee;
- changes in accepted clinical practice that make the continuation of a clinical trial unwise, including the results of similar studies or the publication of international guidances.

2.10 Protocol Amendments

Substantial amendments (significant changes) are only implemented after approval of the CEC and CA respectively.

Significant changes to be authorised by the CEC are the following:

- changes affecting the participants' safety and health, or their rights and obligations;
- changes to the protocol, and in particular changes based on new scientific knowledge which concern the trial design, the method of investigation, the endpoints or the form of statistical analysis;
- a change of trial site, or conducting the clinical trial at an additional site; or
- a change of sponsor, coordinating investigator or investigator responsible at a trial site.

Significant changes to be authorised by Swissmedic are the following:

- changes to the therapeutic product, or to its administration or use;
- changes based on new preclinical or clinical data which may affect product safety; or
- changes concerning the production of the therapeutic product which may affect product safety.

Under emergency circumstances, deviations from the protocol to protect the rights, safety and well-being of human participants may proceed without prior approval of the sponsor and the CEC/CA. Such deviations shall be documented and reported to the sponsor and the CEC/CA as soon as possible.

All non-substantial amendments are communicated to the CA as soon as possible if applicable and to the CEC within the Annual Safety Report (ASR).

3 INTRODUCTION

Coronavirus disease (COVID-19) has emerged as a pandemic and a public health crisis of global proportions. As of April 9, 2020, a total of 1,716,371 COVID-19 cases have been diagnosed with the severe acute respiratory syndrome corona virus 2 (SARS-CoV-2) across more than 170 countries. Of 1,223,520 active cases, 1,173,564 are in a mild and 49,956 are in a serious or critical condition. Of 492,851 closed cases, 103,847 have died and 389,004 recovered or were discharged from hospital (Source: WHO Daily Briefing on COVID-19).

Official data on fatality vary across countries due to differences in the age structure of populations, number of screening tests performed, local capacities of healthcare facilities, and criteria used for the compilation of vital certificates reporting the underlying causes of death. Comparisons with historical data in some European countries suggest, however, that the mortality may be at least four times higher than what is estimated based on official COVID-19 reports, indicating that a substantial number of deaths occurred at home and were not classified as COVID-19-related (1).

In Switzerland, the vast majority of COVID-19-related deaths have been recorded in patients aged 50 years or older with age-dependent mortality and hospitalization rates: <0.5% mortality and 9% hospitalization rate between 50-59 years, 2% and 17% between 60 and 69 years, 7% and 30% between 70 and 79 years, and 16% and 30% above 80 years, respectively.

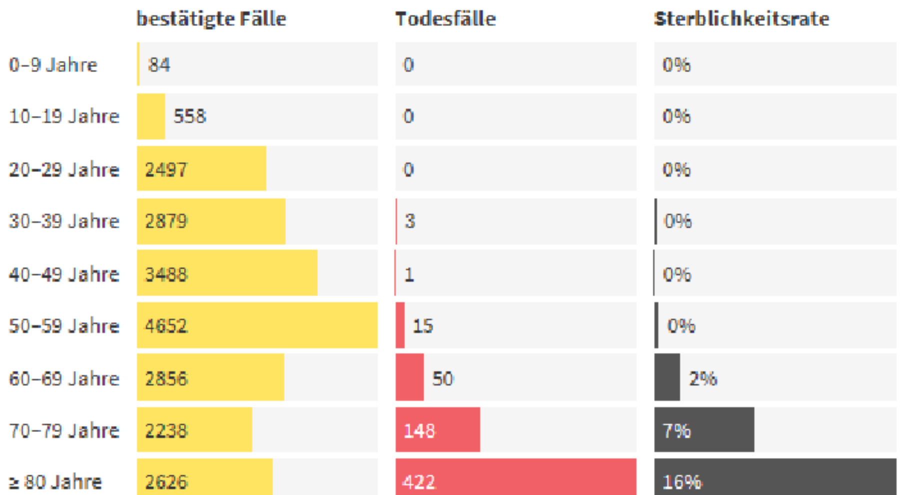

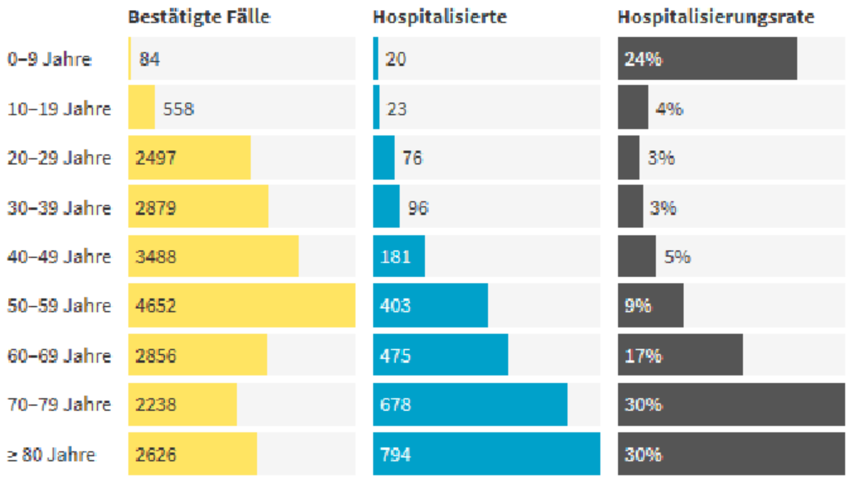

Grafik: pvo • Quelle: BAG • Daten herunterladen

3.1 Background and Rationale

COVID-19 and coagulopathy

One of the most frequently described poor prognostic features in patients with COVID-19 is the development of coagulopathy (2). An increase in D-dimers, which reflects the presence of disseminated intravascular coagulation, is usually observed in the most severe cases of COVID-19 and it correlates with in-hospital mortality (2-4). In hospitalized patients with sepsis-induced coagulopathy or with markedly elevated D-dimer associated with COVID-19 infection, anticoagulation with low-molecular-weight heparin (LMWH) was associated with lower mortality; this association was stronger for higher D-dimer levels: odds ratio for anticoagulation 0.81 (0.48-1.38) for D-dimer > 2 upper limit of normal (ULN) (32.1% vs. 36.9% 28-day mortality), 0.44 (0.23-0.87) for D-dimer > 6 ULN (32.8% vs. 52.4% 28-day mortality). (4)

Although non-survivors with COVID-19 had higher D-dimer levels than survivors, D-dimer has not been studied as a potential predictor of death or VTE events as it represents an non-specific marker. Indeed, the increased levels may reflect acute fibrinous and organising pneumonia characterised by extensive intra-alveolar fibrin deposition (5). Its potential use as a surrogate marker of a prothrombotic condition is in its infancy and

cannot serve to drive medical decisions i.e. on whether to administer anticoagulation or a higher dosage of antithrombotic prophylaxis. Neither the measurement of D-dimers nor the screening with ultrasound are currently recommended in COVID-19 outpatients by international guidelines to provide the indication for thrombosis prophylaxis (6). There are no validated cut-off values for the introduction of D-dimer for risk assessment in COVID-19 and the vast majority of COVID-19 studies adopted incorrect methodology to build prediction models, also concerning the use of D-dimer (7).

In a recent COVID-19 consensus paper, it is stated that “elevated D-dimer levels, is a common finding in patients with COVID-19, and does not currently warrant routine investigation for acute VTE in absence of clinical manifestations or other supporting information.”(6)

COVID-19 and venous thromboembolism (VTE)

COVID-19 emerged as a dramatic prothrombotic condition and it appears now clear, a few months after the first patients described in China, that COVID-19 patients are characterized by a substantial risk of developing acute pulmonary embolism (PE) or deep vein thrombosis (DVT) due to local and systemic inflammation, reduced mobility, hypoxia, endothelial dysfunction. During prior viral outbreaks, such as in patients with Severe Acute Respiratory Syndrome (SARS), myocardial infarction and venous thromboembolism were frequent causes of death (8). These results emerged from clinical studies and post-mortem examinations (recently reviewed in (9)). Small reports on influenza A (H1N1) patients requiring intensive care unit (ICU) admission and advanced mechanical ventilation indicated that PE represented a common finding (10-12).

Published, peer-reviewed reports in the biomedical literature have been consistently showing that DVT and, especially, PE represents a key manifestation of COVID-19 (13-19). We have provided here a brief chronological summary of the most recent evidence that is now growing almost on a daily basis.

Klok et al. (Netherlands)

An analysis of 180 patients admitted at intensive care units in the Netherlands showed that thromboembolic events occurred in 16%, of whom the majority had symptomatic VTE. PE was the most frequent thrombotic complication (n = 25). Age (adjusted hazard ratio (aHR) 1.05/per year, 95%CI 1.004-1.01) and coagulopathy, defined as spontaneous prolongation of the prothrombin time > 3 s or activated partial thromboplastin time > 5 s (aHR 4.1, 95%CI 1.9-9.1), were independent predictors of thrombotic complications (20). These results suggested that a higher dosage of LMWH thromboprophylaxis may be needed in ICU patients due to their extremely high thrombotic risk.

Cui et al. (China)

In 81 ICU patients with COVID-19 diagnosed in China, the incidence of VTE was **25%**: of 20 patients with VTE, 8 died (21). There was no routine screening for VTE in this study, whereas the use of thromboprophylaxis was not clearly documented.

Lltjos et al. (France)

Systematic assessment of VTE using complete duplex ultrasound was performed in anticoagulated COVID-19 patients admitted to 2 French intensive care units (ICU) (17). The overall rate of VTE in patients was **69%**. The proportion of VTE was significantly

higher in patients treated with prophylactic anticoagulation when compared to the other group.

Lodigiani et al. (Italy)

In a recent analysis of 388 Italian hospitalized patients with COVID-19 novel and relevant data emerged. First, a total of 44 (11% of total) patients underwent VTE imaging tests and 16 were positive (**36%** of tests), suggesting substantial underestimation of thromboembolic complications in these patients. Second, half of these events were diagnosed on admission, indicating that VTE did not develop during hospitalization, but in the ambulatory setting (14). Therefore, the key question would not (only) be whether hospitalized COVID-19 patients should receive a higher dosage of LMWH thromboprophylaxis during hospitalization, but if current ambulatory thromboprophylaxis strategies are adequate (22-25).

Helms et al. (France)

A recent analysis from a French group showed that the rate of thromboembolic complications in 150 COVID-19 patients with ARDS was much higher **(11.7%)** than what observed in a historical control group of non-COVID-19 ARDS patients **(2.1%)**, despite anticoagulation (26).

Thomas et al. (UK)

In a British cohort study of 63 ICU patients, the cumulative incidence estimate of VTE was **27%**. However, only 11 patients received CTPA, of whom 5 were positive for PE (18).

Poissy et al. (France)

The authors of a French study (27) showed that the cumulative incidence of PE was **20%** among ICU patients and events occurred after a median of 6 days after hospital admission with 25% of events diagnosed during the first 24 hours.

Middeldorp et al. (Netherlands)

The authors investigated the incidence of objectively confirmed venous thromboembolism (VTE) in 198 hospitalized patients with COVID-19 in a single-center cohort study. Seventy-four patients (37%) were admitted to the intensive care unit (ICU). During a median follow-up of 5 days (IQR, 3-9), 33 patients (**17%**) were diagnosed with VTE of whom 22 (11%) had symptomatic VTE, despite routine thrombosis prophylaxis. The cumulative incidences of VTE at 7 and 14 days were 15% (95% CI, 9.3-22) and **34%** (95% CI, 23-46), respectively. **VTE was associated with death (adjusted HR, 2.9; 95% CI, 1.02-8.0)**. Future research should focus on optimal diagnostic and prophylactic strategies to prevent VTE and potentially improve survival. The authors concluded that "Future research should focus on optimal diagnostic and prophylactic strategies and assessing the risk of VTE in post-discharge and *non-hospitalized patients* with COVID-19." Preprint available at: https://www.preprints.org/manuscript/202004.0345/v1.

Taking this information into account, it is clear that COVID-19 represents an unprecedented and highly prothrombotic condition for which current thromboprophylaxis schemes may be inadequate and novel strategies are warranted. Only well-conducted randomized controlled trial can provide physicians with useful information to guide clinical decisions.

Risk of VTE during other viral infections or medical illnesses

Data from the pre-COVID-19 era indicate that flu and other seasonal viral infections increase the risk of developing VTE in the general population, but do not represent per se an indication to give thromboprophylaxis in the outpatient setting (28). No large interventional studies have ever been conducted in the outpatient setting to investigate this point. Indeed, there are two key differences with the COVID-19 outbreak:

1) The vast majority of people aged 60 or older (as well as other "fragile" population subgroups) are routinely vaccinated against influenza, the most feared and prevalent respiratory infection in the elderly. This is obviously not the case for SARS-CoV2.
2) The burden of viral respiratory infection-associated VTE, at least based on indirect data from hospitalized patients, does not appear to be dramatic or extreme as among COVID-19 patients.

There are further methodological and epidemiological aspects to consider:

- the field of viral infection-associated thromboembolism is in its infancy: although up to 7% of unprovoked VTE events may be due to viruses, e.g. cytomegalovirus, (29) this patient population has not been object of focused investigations;

- there is data from case-control studies informing us about the prevalence of prior (or recent) influenza infection in patients with vs. without acute VTE, but evidence on the absolute risk of VTE among patients with a respiratory/influenza infection is less firm and usually concerning hospitalized patients (30);

- approximately 75% of acute VTEs occur in the outpatient setting and acute respiratory infection is one of the highest prevalent risk factor after cancer and recent hospitalization/surgical intervention.

It is known from the literature that hospitalized non-ICU patients with medical illnesses receiving adequate thromboprophylaxis are characterized by rates that are close to 0% during the first 2 weeks of hospitalization, and indeed not exceeding 3% (31-33).

COVID-19 and cardiovascular events

COVID-19 patients with chronic coronary artery disease and those with risk factors for atherosclerotic cardiovascular disease have an increased risk to develop myocardial infarction during acute infections, as shown previously in epidemiologic and clinical studies of influenza (34, 35). Acute cardiac injury was reported in 12% of COVID-19 cases in a small case series in *The Lancet*. Another study suggested a rate of 7.2% among 138 patients from another hospital in Wuhan (36). Shi et al. and Guo et al. showed that patients with myocardial injury had a higher prevalence of hypertension, coronary artery disease, heart failure, and diabetes, as well as more severe inflammation, than those with normal levels of troponin (37, 38). In China, COVID-19 patients with signs of myocardial injury had much higher short-term mortality rates (37, 38).

As with other coronaviruses, SARS-CoV-2 can elicit the intense release of multiple cytokines and chemokines that can lead not only to vascular inflammation and plaque instability but also to myocardial inflammation (35). The possibility of direct viral infection of vascular endothelium and myocardium via the host ACE-2 receptor has also been raised (39).

The USZ was largely involved in the study of the pathophysiology and complications of COVID-19 (14, 40), and identification of key pathophysiological mechanisms:

1) Coronavirus binds to the ACE-2 respiratory receptor and causes a respiratory infection,

2) Coronavirus rejection via the lungs into the bloodstream,

3) Coronavirus binds on the ACE-2 receptors of the endothelium and penetrates the endothelial cells and causes severe endothelitis in many organs with high amounts of receptors (virus direct detection in the endothelium),(40)

4) Coronary virus causes severe endothelitis that we do not see in other viral diseases,(40)

5) «Extreme» coagulation activation and coagulopathy, measurable and strongly increased fibrinogen and D-dimers,

6) VTE, micro-thrombosis also in other organs, cardiovascular events (2, 6, 14).

Thromboprophylaxis

A recent ISTH SSC guidance stated that prophylactic-dose LMWH, such as enoxaparin, should be considered in all patients who require hospital admission for COVID-19 in the absence of any contraindications (41). One of the better known non-anticoagulant properties of heparins, their anti-inflammatory function, include binding to inflammatory cytokines, inhibition of neutrophil chemotaxis and leukocyte migration, neutralization of the positively charged peptide complement factor C5a, and sequestering acute phase proteins: this may provide a benefit in COVID infection where proinflammatory cytokines are markedly raised and acute respiratory distress syndrome represents a feared and life-threatening complication (41, 42). A paper published in 1992, has also shown that heparin can reduce myocardial inflammation and decrease collagen deposition in an animal model of (chronic) myocarditis (42, 43), a frequent finding in COVID-19 patients. Furthermore, in vitro experiments showed that LMWH may be characterized by a protective effect against SARS-CoV and SARS-CoV2 viruses, particular limiting the SARS-CoV1 invasion at the early attachment phase during the initial phase and inhibit virus cell invasion (44).

According to international consensus documents and guidelines, LMWH thromboprophylaxis should be considered in most hospitalized patients with medical illnesses. Currently, there is no firm evidence that LMWH thromboprophylaxis should be routinely used in ambulatory medical patients.

In Switzerland, as in many other European countries, LMWH is approved for thromboprophylaxis in patients with bed rest and acute medical illnesses, such as (i) heart failure, (ii) acute respiratory insufficiency, or (iii) acute infectious or rheumatological conditions in combination, the latter, requiring an additional risk factor or immobilization.

A practical guidance for the prevention of thrombosis and management of coagulopathy and disseminated intravascular coagulation of patients infected with COVID-19 stated that the risk of VTE must be assessed in all patients admitted to hospital, and preventive measures should be taken in all high-risk patients according to international guidelines on thromboprophylaxis in medical patients (NICE/ASH) (45). Several scientific societies, such as the American Society of Hematology, Thrombosis UK, ISTH, and the Gesellschaft für Thrombose- und Hämostaseforschung (GTH), recommend the use of

LMWH over new oral anticoagulants for thromboprophylaxis in COVID-19 patients in the presence of VTE risk factors. Of note, bleedings rarely occur in patients with COVID-19 (42). However, the lack of firm evidence prevents the release of more specific recommendations.

A recent review on the topic addressed this point (46) with a general statement "While routine use of thromboprophylaxis in outpatients is not recommended, use in immobile infected outpatients, especially with other increased risks for VTE, can be considered on a case by case basis based on severity of illness or as incorporated into local practice".

Taking into accounts all these factors, anticoagulant prophylaxis is being used in the vast majority of COVID-19 patients admitted to the hospital as the thromboembolic risk of these patients is perceived to largely exceed that of bleeding.

Due to the substantial societal burden posed by this outbreak, most Western countries developed strategies to handle as many patients as possible on an ambulatory basis. Admitted COVID-19 patients represent a minority, even among the elderly. Ambulatory treatment includes testing, general assessment of the vital parameters, blood withdrawal, and supportive therapy. Although there is heterogeneity across regions and countries, quarantine represents the most adopted public health measure to limit viral transmission by isolating patients positive for SARS-CoV2.
It remains unclear whether patients not admitted to the hospital due to non-severe clinical conditions should receive thromboprophylaxis and whether this provides a clinical benefit weighed against the risk of anticoagulant-associated bleeding. The evidence is scarce also for non-COVID-19 patients. The most recent American Society of Hematology (ASH) guidelines state that "In medical outpatients with minor provoking risk factors for VTE (eg, immobility, minor injury, illness, infection), the ASH guideline panel suggests not using VTE prophylaxis (conditional recommendation, very low certainty in the evidence of effect)."
However, it may be dangerous to extrapolate this low-evidence-level recommendations to patients who may be characterized by a substantial burden of VTE due to coagulation activation, local and systemic inflammation, reduced mobility (42).

Rationale
The following points represent, in summary, the rationale for studying the use of thromboprophylaxis in ambulatory patients with COVID-19:

1) The risk of thromboembolic events in patients with COVID-19 during anticoagulant prophylaxis exceeds that observed in medical patients, usually <3%, even in the presence of seasonal viral infections (47, 48)
2) The cumulative risk of VTE in hospitalized COVID-19 patients is at least 20%, but possibly higher, as described in several publications
3) The absolute VTE risk in COVID-19 patients requiring intensive care is 69% if screening strategies are implemented (17)
4) Half of the VTE events, mostly PE, were diagnosed at hospital admission, suggesting that these events developed during the quarantine period (14).

Our hypothesis is that early thromboprophylaxis may prevent or limit coagulopathy, and reduce thromboembolic complications leading to hospitalization or death, in the presence of a mild COVID disease among outpatients.

3.2 Investigational Product and Indication

Enoxaparin sodium is a low molecular weight heparin marketed under the trade names Clexane® and associated names. This anticoagulant is used in the treatment and prophylaxis of thromboembolic disorders. It is given as an injection subcutaneously or intravenously.

The principal pharmacological properties of enoxaparin include antifactor Xa and antifactor IIa (antithrombin) activity, which are dependent on its binding affinity for antithrombin. Clexane® and associated names solution for injection is currently approved in more than 140 countries worldwide including all the European Union (EU) member states as well as Switzerland. The first marketing authorisation (MA) was granted in France on 03 April 1987.

The product is currently registered in Europe under concentrations of 100 mg/mL (equivalent to 10 000 IU anti Xa/mL) in prefilled syringes, multi-dose vials, ampoules, and 150 mg/mL (equivalent to 15 000 IU anti Xa/mL) in prefilled syringes. Vials of 100 mg/10 mL and a pen of 10 x 40 mg (equivalent to 10 x 4 000 IU anti Xa) are also authorised. One mg of enoxaparin exhibits 100 IU anti-Xa, this allows an easy conversion and representation of the anti-Xa activity for the prescriber, and referring to mg instead of biological activity units has been commonly used throughout clinical trials.

Overall, the general principles of prophylactic and therapeutic indications of enoxaparin are reflected in the PIs across Europe, however the section was harmonised to address variations in the exact wording in these types of indication, which were different between States.

Enoxaparin is indicated in adults for:

• Prophylaxis of venous thromboembolic disease in moderate and high risk surgical patients, in particular those undergoing orthopaedic or general surgery including cancer surgery.

• Prophylaxis of venous thromboembolic disease in medical patients with reduced mobility and an acute illness (such as acute heart failure, respiratory insufficiency, severe infections or rheumatic diseases at increased risk of venous thromboembolism).

• Treatment of deep vein thrombosis (DVT) and pulmonary embolism (PE), excluding PE likely to require thrombolytic therapy or surgery.

• Prevention of thrombus formation in extracorporeal circulation during haemodialysis.

• Acute coronary syndrome:

- Treatment of unstable angina and Non ST-segment elevation myocardial infarction (NSTEMI), in combination with oral acetylsalicylic acid.

- Treatment of acute ST-segment elevation myocardial infarction (STEMI) including patients to be managed medically or with subsequent percutaneous coronary intervention (PCI).

There are few differences in the various country labels regarding the indication prophylaxis of VTE in medical patients. While e.g. the term “bedridden” appears in many countries as a key criteria, some countries have more open definition such as “whose position can be defined at risk” or “temporary immobilised”.

The ACCP guidelines recommend: For acutely ill hospitalised medical patients at increased risk of thrombosis, we recommend anticoagulant thromboprophylaxis with LMWH, low-dose unfractionated heparin (LDUH) twice a day (BID), LDUH three times a day (TID), or fondaparinux: Grade 1B.

The revised wording is based on the inclusion criteria and the results of the MEDENOX study (31). The harmonised wording for the indication in this population therefore includes “disease in medical patients with an acute illness (such as acute heart failure, respiratory insufficiency, severe infections or rheumatic diseases) and reduced mobility at increased risk of venous thromboembolism”.

In Switzerland, enoxaparin is approved for the same prophylactic indications. In medical patients with acute illness, the recommended dosage is 40 mg qD s.c. given as a subcutaneous injects for a period of 6-14 days, until complete mobility is not reached. The benefit is not established for a treatment longer than 14 days.

From the Swiss product monograph (Swissmedic-genehmigte Fachinformation):

«Prophylaxe von thromboembolischen Erkrankungen venösen Ursprungs bei bettlägerigen medizinischen Patienten mit einer akuten Erkrankung, wie: Herzinsuffizienz (NYHA Klasse III oder IV); Akute respiratorische Insuffizienz; Akute infektiöse oder rheumatische Erkrankungen verbunden mit einem/anderen thromboembolischen Risikofaktor/en.»

In acutely ill medical patients, the American Society of Hematology (ASH) guideline panel suggests using unfractionated heparin, LMWH, or fondaparinux rather than no parenteral anticoagulant (conditional recommendation, low certainty in the evidence of effects). Among these anticoagulants, the panel suggests using LMWH (low certainty in the evidence of effects) or fondaparinux (very low certainty in the evidence of effects) rather than UFH (conditional recommendation).

3.3 Preclinical Evidence

Besides the anticoagulant effects of enoxaparin sodium, there was no evidence of adverse effects at 15 mg/kg/day in the 13-week SC toxicity studies both in rats and dogs and at 10 mg/kg/day in the 26-week SC and IV toxicity studies both in rats, and monkeys.

Enoxaparin sodium has shown no mutagenic activity based on in vitro tests, including the Ames test, mouse lymphoma cell forward mutation test, and no clastogenic activity based on an in vitro human lymphocyte chromosomal aberration test, and the in vivo rat bone marrow chromosomal aberration test.

Studies conducted in pregnant rats and rabbits at SC doses of enoxaparin sodium up to 30 mg/kg/day did not reveal any evidence of teratogenic effects or foetotoxicity. Enoxaparin sodium was found to have no effect on fertility or reproductive performance of male and female rats at SC doses up to 20 mg/kg/day.

3.4 Clinical Evidence to Date and post-market experience

Enoxaparin has been used for more than 20 years and represented the reference treatment for the large majority of phase III trials studying direct oral anticoagulants (rivaroxaban, dabigatran etexilate, apixaban, edoxaban) for the prevention of VTE in medical (49) and surgical orthopaedic patients (50) and for the acute treatment of VTE (51), including cancer-associated VTE (52) and pregnancy-associated VTE (53). It remains one of the treatments of choice for these indications (54-57).

3.5 Dose Rationale

The proposed dose, 40 mg/day, and treatment duration (14 days) is in line with the product label, which are primarily based on the results from the MEDENOX study (58) where this was effective, as opposed to the 20 mg/day dose that was not. The MEDENOX study evaluated enoxaparin at both 2000 UI and 4000 UI. Only enoxaparin at 4000 UI demonstrated the efficacy for the prevention of VTE in medical patients. In addition, the mean duration of treatment is 10 days, up to 14 days in the MEDENOX study. The harmonised treatment duration is aligned with the data from the MEDENOX study (6 to 14 days), as there is no evidence that a shorter duration may be effective (58).

3.6 Explanation for Choice of Comparator

Risk assessment models (i.e. the PADUA and the IMPROVE scores) are used to identify patients who should receive in-hospital thromboprophylaxis based on a risk benefit assessment according with international guidelines (57). Enoxaparin may be used in ambulatory patients with acute infectious illness, if an additional VTE risk factor is present. Therefore, no treatment represents the current standard of care for the COVID (+) patients without additional VTE risk factor.

3.7 Risk/benefits

Published data and studies under review (co-authors are the OVID investigators) suggest that (i) COVID-19 is highly prothrombotic, (ii) a significant number of VTE events occur in ambulatory patients and is detected at the time of initial evaluation. Therefore, higher-dosage thromboprophylaxis has been postulated for admitted patients and routine thromboprophylaxis may be useful for ambulatory patients in order to prevent potential thromboembolic complications before the patients´ general conditions precipitate.

Enoxaparin is effective in preventing VTE in medical patients with acute illness. Its anti-inflammatory and antithrombotic profile may be highly beneficial in the setting of COVID-19. Initial report in admitted COVID-19 patients shows that LMWH appears to be beneficial in the presence of coagulation activation (4).

The ambulatory administration of injectable anticoagulants, including LMWH and the pentasaccharide fondaparinux, has been and is standard of care for a number of clinical conditions, including thromboprophylaxis after major orthopedic surgery and, especially in the era preceding the approval of direct oral anticoagulants, also the treatment of acute VTE. Indeed, the initial use of LMWH remains standard of care for patients, especially

the elderly, with contraindications to the use of direct oral anticoagulants. In this setting, patient instructions are usually given during the initial in-hospital visit, corresponding to the day of hospital discharge after orthopedic surgery or the day of acute VTE diagnosis; subsequently, patients self-administer the prefilled anticoagulant injections, alone or with the help of family members, in the ambulatory setting and, depending on the indication, for a period of 10-35 days (i.e. 35 days after hip arthroplasty, 14 days after knee arthroplasty). Of note, 6-month (ambulatory) therapy with subcutaneous LMWH still represents the therapy of choice of the treatment of acute VTE among several groups of cancer patients (59).

In-hospital follow-up visits after the start of prophylactic-dose anticoagulant administration were not part of the study procedures of recent orthopedic trials on thromboprophylaxis, which usually included early phone follow-up intermediate to verify compliance and the potential occurrence of adverse events (60, 61).

Enoxaparin is characterized by a high safety profile and has been used for multiple indications for over 20 years. A recent meta-analysis of thromboprophylaxis trials in medically ill patients showed that a major bleeding event occurred in 157 of 26608 (0.6%) of patients during short-course administration (14 days of lower). The two largest and most recent phase III trials on extended thromboprophylaxis in medically ill patients, the MAGELLAN and MARINER study, had major bleeding rates of 0.3% and 0.2%, respectively (32, 62).

It must be further noted that approximately 1% of the general population receive therapeutic-dose anticoagulant treatment for the prevention of embolic events due to atrial fibrillation or for the treatment of VTE. This prevalence of anticoagulant users exceeds 3-5% among the elderly. Anticoagulant treatment is one of the best-known medical therapies used in Western countries and the management of potential complications is part of routine clinical practice.

The risk of heparin-induced thrombocytopenia in LMWH-treated patients is negligible (0.8%) and of those who develop heparin-induced thrombocytopenia only a minority would develop a thromboembolic event (30% of 0.8% = 0.024% of patients receiving LMWH) (63).

As reported in Section 8.1.1. enoxaparin does not accumulate in the body after multiple administrations in the absence of severe renal dysfunction, one of the key exclusion criteria of the OVID study.

Moreover, the study will include only patients in whom the self-administration of enoxaparin (or the administration by a person living in the same house) after instructions is deemed possible (see Inclusion Criteria). This is in line with current practice, e.g. after orthopaedic surgery or in cancer patients with acute VTE.

Currently, in Switzerland there is no specific follow-up strategy for patients who are tested positive for SARS-CoV2 and are candidates to an ambulatory treatment.

The current standard of care for outpatient COVID treatment at some centers is as follows:

1) Patient goes to the test center with symptoms;

2) The physician performs a SARS-CoV2 test and triage the patient concerning potential hospitalization. This may include standard assessment of the presence of DVT or PE, if

deemed necessary. If not, the patient is discharged to home quarantine without taking blood and without performing any laboratory diagnostics.

3) There is no direct contact with doctors and care in the home quarantine in order not to endanger personnel.

OVID may increase the level of safety compared to standard of care for study patients because frequent telephone visits are planned (Day 3, 7, 14, 30, 90). This regards also patients who are not randomized to the intervention arm. This approach is now becoming standard of care at some COVID-19 centers: however, this is not uniform across the country and the planned contacts are less frequent than in the OVID study. Thus, being part of a clinical study with predefined phone contacts will allow the patient to be constantly followed by medical and study personnel. The standardized visit questionnaire ensures that relevant bleeding is not missed.

Ethical considerations
There is substantial uncertainty regarding the routine use of thromboprophylaxis for COVID-19 in the outpatient setting. As previously discussed, recommendations are based on expert consensus and are largely adapted on local protocols.
The present study hypothesizes a potential benefit of thromboprophylaxis, which could reduce the number of hospitalizations (primarily due to thromboembolic complications) and the number of deaths among enoxaparin-treated patients.

The thromboembolic manifestations of COVID-19 have been object of extensive media coverage. Therefore, we anticipate that patients may be worried of being randomized to the control group and not receiving anticoagulant prophylaxis. The investigators will make clear during the patient consent procedure that (i) the potential benefits of thromboprophylaxis have not been demonstrated yet, (ii) the use of enoxaparin is characterized by a low (and well-quantifiable from prior studies) risk of major bleeding, <1% within 2 weeks of treatment, (iii) the risk benefit balance should be objective of investigation in the context of a phase III trial, as there is no current evidence supporting the routine use of thromboprophylaxis.

3.8 Study population

The study population was selected based on the following reasons:

- COVID-19-patients 50 years or older are those characterized by the highest hospitalization and fatality rates and, therefore, those who may benefit most from a prophylactic therapy with enoxaparinWe will include only symptomatic patients who presented with fever or respiratory symptoms, therefore aiming to minimize the number of incidental positive tests done for screening reasons.Patients at the highest risk of VTE, including patients with cancer or with prior VTE, will be excluded as it would not be appropriate to include those, as the authors believe that they should routinely receive ambulatory thromboprophylaxis.
- At the same time, patients characterized by a high bleeding risk will be excluded, including those with intracranial malignancy, recent bleeding, or strong dual antiplatelet therapy.
- Recent data suggest that several VTE and deaths are recorded during “secondary” hospitalization which followed a diagnosis of COVID-19 and an initial ambulatory

treatment.

- The almost totality of COVID-19 trials is currently focusing on hospitalized patients: therefore, our study will not be competing with other studies for enrolment;
- Of note, patient with an initial suspicion of symptomatic VTE (i.e. upon SARS-CoV2 testing) will and should undergo standard assessment by VTE imaging and, in the case that VTE is confirmed, the patient cannot be considered eligible for inclusion in the OVID study. The screening strategies upon SARS-CoV2 testing are left at the discretion of the treating physicians as at the moment there is no evidence that those should be implemented.

4 STUDY OBJECTIVES

4.1 Overall Objective

The OVID study will show whether enoxaparin improves survival and reduces hospitalization in ambulatory patients aged 50 or older diagnosed with COVID-19, a novel viral disease characterized by severe systemic, pulmonary, and vessel inflammation and coagulation activation.

4.2 Primary Objective

The purpose of this study is to show the superiority of ambulatory thromboprophylaxis with enoxaparin versus no treatment to prevent any hospitalization and all-cause death within 30 days of randomization in patients aged 50 or older with COVID-19.

4.3 Secondary Objectives

Key secondary objectives for this study are to determine if enoxaparin administration versus no treatment reduces specific cardiovascular and thromboembolic complications, namely venous thromboembolism, myocardial infarction or stroke, within 14 days, 30 days and 90 days of randomization, and if this intervention is associated with a net clinical benefit, accounting for major bleeding events.

Subgroup analysis to study treatment effects of enoxaparin (versus no treatment) will be conducted in specific subgroups of patients categorized by sex, age (50-70 vs. >70 years), renal function (estimated renal function <50 ml/min vs. 50 ml/min or higher), and concomitant antiplatelet therapy.

4.4 Safety Objectives

The study will assess the safety of thromboprophylaxis in COVID-19 ambulatory patients aged 50 or older and quantify the risk of major bleeding, non-major clinically relevant bleeding, and adverse events.

5 STUDY OUTCOMES

5.1 Primary Outcome

A composite of any hospitalization or all-cause death occurring within 30 days of randomization.

5.2 Secondary Outcomes

1) Composite outcome of cardiovascular events, including deep vein thrombosis (including catheter-associated), pulmonary embolism, myocardial infarction/myocarditis, arterial ischemia including mesenteric and extremities, acute splanchnic vein thrombosis, or ischemic stroke within 14 days, 30 days, and 90 days of randomization

1) Each component of the primary efficacy outcome, within 14 days, 30 days, and 90 days of randomization.

2) Net clinical benefit (accounting for the primary efficacy outcome, composite cardiovascular events, and major bleeding), within 14 days, 30 days, and 90 days of enrolment.

3) Primary efficacy outcome, within 14 days, and 90 days of enrolment.

4) Disseminated intravascular coagulation (ISTH criteria, in-hospital diagnosis) within 14 days, 30 days, and 90 days of enrolment.

Important cardiovascular events have been listed as secondary outcomes as we anticipate that appropriate imaging tests may be underused in this population, leading to an underestimation of these rates in both groups. In light of the above, information on secondary outcomes will be retrieved from medically certified discharge letters and ambulatory/laboratory reports, therefore not requiring formal event adjudication.

5.3 Other Outcomes of Interest

Not applicable.

5.4 Safety Outcomes

The principal safety outcome is major bleeding, defined as overt bleeding associated with a decrease in the hemoglobin level of 2 g/dL (1.24 mmol L^{-1}) or more, bleeding that led to transfusion of 2 or more units of packed red cells or whole blood, bleeding that occurred in a critical site (i.e., intracranial, intraspinal, intraocular, pericardial, intraarticular, intramuscular with compartment syndrome, or retroperitoneal), or fatal bleeding (ISTH criterion) (64).
The other safety outcome is non-major clinically relevant bleeding and adverse events. Non-major clinically relevant bleeding is defined as overt bleeding that did not meet the criteria for major bleeding but was associated with medical intervention, unscheduled

contact (visit or telephone call) with a physician, temporary cessation of the trial regimen, or pain or impairment of activities of daily life (ISTH criterion) (64).
All serious adverse events potentially related to the study medication (i.e. local and systemic bleeding complications, allergic reactions) and reasons for hospitalizations except the study outcomes (VTE-related death, myocardial infarction, stroke, symptomatic VTE [DVT and PE], bleeding events) will be collected.

6 STUDY DESIGN AND COURSE OF STUDY

6.1 General Study Design and Justification of the Design

The study will be conducted as a multicentre randomized open-label controlled trial. In the study, a total of 1,000 adult patients aged 50 or older with symptomatic COVID-19, without other indications to anticoagulant therapy, and candidates to ambulatory treatment will be randomized to receive enoxaparin 40 mg sc qD or no treatment for a total of 14 days. The primary outcome is any hospitalization or all-cause death within 30 days of enrolment. A final 90-day phone contact is planned.

6.2 Study Duration and Study Schedule

Patient enrolment will last up to 6 months and will take place at 7 Swiss centers, including five university hospitals and two large cantonal hospitals. The study period will therefore consist of a total of approximately 10 months.

Study schedule:

1.	Submission to HA/EC	06/05/2020
2.	First Patient, First Visit date:	25/05/2020
3.	First Patient Last Visit date:	14/10/2020
4.	Last Patient Last Visit date:	14/03/2021
5.	Report(s) date	14/04/2021
6.	Planned Publication(s) date	14/05/2021

6.3 Methods of Minimising Bias

6.3.1 Randomisation

Patients will undergo block stratified randomization (by age 50-70 vs. >70 years and study center) with a randomization ratio of 1:1 with block sizes varying between 4 and 8.

6.3.2 Blinding Procedures

No blinding procedures will be used in this study for logistical reasons.

6.3.3 Other Methods of Minimising Bias

Predefined questionnaires will serve to guide telephonic contacts with patients during (pre-)screening and follow-up visits. The adoption of objective study efficacy outcomes (any hospitalizations, all-cause death) aims at minimizing the risk of subjective interpretation.

6.4 Unblinding Procedures (Code break)

Not applicable.

7 STUDY POPULATION

An average of 140 patients will be enrolled at each of the 7 study centers, represented by all the university Swiss hospitals and two large cantonal hospitals. It is expected that patients will be enrolled over 6-7 months, corresponding to an average of approximately one patient per day per center. In case the enrolment goals are not met after the first 3 months from study start, the participation of additional centers will be considered, as well as the extension to other European countries upon positive application for public funding.

7.1 Eligibility Criteria

7.1.1 Inclusion Criteria

1) Signed patient informed consent after being fully informed about the study's background.
2) Patients aged 50 years or older with a positive test for SARS-CoV2 in the past 5 days and eligible for ambulatory treatment.
3) Presence of respiratory symptoms (i.e. cough, sore throat, or shortness of breath) or body temperature >37.5° C.
4) Ability of the patient to travel to the study center by private transportation, performed either by accompanying person from same household or by the patient him/herself
5) Ability to comply with standard hygiene requirements at the time of in-hospital visit, including a face mask and hand disinfectant.
6) Ability to walk from car to study center or reach it using a wheel chair transport with the help of an accompanying person from the same household also complying with standard hygiene requirements.
7) Ability to self-administer prefilled enoxaparin injections after instructions received at the study center or availability of a person living with the patient to administer enoxaparin.

7.1.2 Exclusion Criteria

The presence of any one of the following exclusion criteria will lead to exclusion of the participant:

1) Any acute or chronic condition posing an indication for anticoagulant treatment, e.g. atrial fibrillation, prior VTE, acute confirmed symptomatic VTE, acute coronary syndrome.
2) Anticoagulant thromboprophylaxis deemed necessary in view of the patient's history, comorbidity or predisposing strong risk factors for thrombosis:
    a. Any of the following events occurring in the prior 30 days: fracture of lower limb, hospitalization for heart failure, hip/knee replacement, major trauma, spinal cord injury, stroke,
    b. previous VTE,
    c. histologically confirmed malignancy, which was diagnosed or treated (surgery, chemotherapy, radiotherapy) in the past 6 months, or recurrent, or metastatic, or inoperable.
3) Any clinically relevant bleeding (defined as bleeding requiring hospitalization, transfusion, surgical intervention, invasive procedures, occurring in a critical anatomical site, or causing disability) within 30 days prior to randomization or sign of acute bleeding.
4) Intracerebral bleeding at any time in the past or signs/symptoms consistent with acute intracranial hemorrhage.
5) Hemoglobin <8 g/dL and platelet count <50 x 10^9 cells/L confirmed by recent laboratory test (<90 days).
6) Subjects with any known coagulopathy or bleeding diathesis, including known significant liver disease associated with coagulopathy.
7) Severe renal insufficiency (baseline creatinine clearance <30 mL/min calculated using the Cockcroft-Gault formula) confirmed by recent laboratory test (<90 days).
8) Contraindications to enoxaparin therapy, including prior heparin-induced thrombocytopenia and known hypersensitivity.
9) Current use of dual antiplatelet therapy.
10) Participation in other interventional studies over the past 30 days.
11) Non-compliance or inability to adhere to treatment or lack of a family environment or support system for home treatment.
12) Cognitive impairment and/or inability to understand to information provided in the study information.

7.2 Recruitment and Screening

Pre-screening and screening phase

We considered three main screening scenarios preceding the in-hospital baseline visit:

- Scenario 1: Screening at the study center on the day of SARS-CoV2 testing
- Scenario 2: Referral from the patient (via Hot-Line or study center).
- Scenario 3: Referral from COVID testing centers or primary care physicians (via Hot-Line or study center).

<u>Scenario 1: Enrolment at the study center on the day of SARS-CoV2 testing</u>

1) Test for SARS-CoV2: i.e. at the Emergency Room or at COVID testing center at the study site.
2) The patient waits for the results of SARS-CoV2 (i.e. done with rapid tests) at the same center, as per local standard practice in a deputed room.

3) Discussion with the study investigator, signature of PIC2, evaluation of the eligibility criteria. If not available, additional blood tests (blood cell counts, renal function) will be performed as part of the study screening using standard measurement or point of care devices.
4) Details on randomization, allocation, and instructions are given in the next paragraph ("In-hospital visit").

Scenario 2: Referral from the patient.

1) The patient undergoes SARS-CoV2 test and gets to know about the OVID study (Flyer).
2) The patient goes home and waits for the test results being communicated by the test center.
3) The patient is informed by the testing center about his/her positive SARS-CoV2 test.
4) He/she phones the trilingual Hot-Line or directly the referral study center and the potential study participation is evaluated first, particularly concerning the formal OVID eligibility criteria (see pre-screen Questionnaire for Hot-Line and study centers).
5) If the patient is potentially eligible, the Hot-Line personnel or the study center plans a further in-hospital visit at the closest study center within 5 days from positive SARS-CoV2 test.
6) See the paragraph "In-hospital visit" for further details.

Scenario 3: Referral from COVID testing centers or primary care physicians.

1) The patient undergoes SARS-CoV2 test and gets to know about the OVID study (Flyer and PIC1) and potential eligibility.
2) The patient allows the test center personnel to inform the study physicians about (i) his/her positive test and (ii) his/her contact information (signature of PIC1).
3) The patient goes home and waits for the test results being communicated by the test center.
4) The test center informs the Hot-Line or study center about (i) patient's positive test and (ii) his/her personal information.
5) The Hot-Line or study center phones the patient and the potential study participation is further evaluated, particularly concerning the formal OVID eligibility criteria (see pre-screen Questionnaire).
6) If the patient is potentially eligible, the Hot-Line personnel of the study center plans a further in-hospital visit at the closest study center within 5 days from positive SARS-CoV2 test
7) See the paragraph "In-hospital visit" for further details.

Hot-Line procedures

Patient recruitment will be centrally organised and coordinated also with the aid of a national Hot-Line in three languages (German, French, Italian), ensuring a one-time contact with patients to preliminary verify the eligibility criteria and plan an in-hospital visit for screening and recruitment. The Hot-Line number will be published on official websites and will be made available as part of an awareness campaign organized by the communication department of the USZ.

Deputed personnel receiving following precise instructions given by the study investigators will be available during working hours to discuss with patients. A predefined pre-screening Questionnaire will be completed telephonically following a step-by-step standardized procedure. If the patient is not eligible, i.e. one red box is checked in the questionnaire, the questionnaire will be destroyed immediately after compilation by the Hot-Line personnel and no personal data will be registered. If the patient is potentially eligible, a study visit will be planned from the Hot-Line at the closest study center or the study center will be contacted directly by the patient. The Hot-Line will provide the study investigators at each study center with a copy of the Questionnaire containing pre-screening information. The study site is then asked to complete the questionnaire with the data and time of the in-hospital visit and return the questionnaire to the study e-mail address: ovidstudie@usz.ch.

Trained staff for the Hot-Line may include physicians, nurses, medical students, and administrative personell, who is also educated to encourage the patient (i) to bring any medical documents to the in-hospital visit that may facilitate enrolment, e.g. medication lists or medical reports or the results of recent laboratory testing and if applicable a copy of the signed PIC 1. In addition the Hot-Line personnel will provide the study information and consent form (PIC 2) by email if desired by the patient.

The Hot-Line personnel instructs the patient to come by private car, use the reserved parking spaces and stay in the car until he/she gets further instructions from the study personnel. Information concerning the precise location of the parking place at each study center will be given telephonically to the patients and may be sent via e-mail upon patient’s request by local study personnel.

In-hospital visit

1. The study personal reaches the car of the patient and provides him/her and if applicable his/her companion with face masks and desinfectant. The patient will then follow the study coordinator to the assigned study rooms. If the patient is dependent on a wheel chair, he/she must be able to transfer him/herself or with the aid of the companion into the wheel char. The accompanying person is only allowed to enter the study rooms and attend the study visit, if he/she is determined to apply the clexane injections. In this case, the accompanying person gets the clexane administration instruction.
2. The study physician will discuss the scope of the study and answers all questions of the patient. Is the patient willing to participate in the study, he/she will sign the PIC 2. After signature of PIC2, a further check of inclusion and exclusion criteria will be performed by the treating physician, using if available medical documents. A blood test will be performed, if no blood test results are available. During blood analysis, the other parameters for the screening visit can be collected, e.g. vital signs, demographics, etc. (see paragraph study assessment).
3. If the blood test is in line with the inclusion and exclusion criteria, the study physician performs the final check of the eligibility. If eligibility cannot definitively be confirmed, no randomization will be performed and the patient will be counted as screening failure.
4. If eligibility is confirmed, Randomization will be done. The patient receives a study participant card, where the contact data from the study team are reported and information on study drug allocation.

5. For patients from the control group, patient will return home by private car transport. For patients form the enoxaparin group, instruction for use of enoxaparin injections will be provided. For interested patients, a link for a video instruction will be provided. The first injection of study drug will be performed at study center, if possible by the patient. If patient does not inject the study drug, the accompanying person gets the instruction and will perform the first injection.
6. For patients of the treatment group, the required amount of study drug will be provided to the patients with instructions for documentation. The patients are encouraged to fill in the study diary, which is provided by the study team and will be instructed to take note of doses that are forgotten or missed. This form will be handed to the patients with a pre-stamped reply covert to simplify the return process.

The study visit is planned for at least 60 minutes for all patients who received the OVID patient information by email in advance. The study visit is planned for at least 90 minutes for all patients who did not receive the OVID patient information by email in advance to provide enough time for considering study participation.

Only one in-hospital visit is planned. As previously described, the best care will be taken to minimize the discomfort for the patient and maximize safety for the investigators. To reduce the infection risk, only a study physician will be in the study room with the patient.

If needed, the blood test (hemoglobin, platelet count, creatinine) may be performed by the use of a point-of-care device, which guarantees a result within minutes and the study physician will be informed about the results immediately.

All the participating centers agreed to fulfil these logistic requirements and will be able to guarantee the same standard of care by signing a contract. These requirements were part of the criteria for the selection of the study sites.

Screening failures

Definition:

All patients who signed the informed consent 2 (PIC2) but were not eligible for randomization.

Documentation

A screening log will be filled out at each study center, listing all patients with date and reason of screening failure. This document will be signed by the local principal investigator at the end of the recruitment period and returned to the sponsor.

Patient and Public Involvement

These recruitment and study procedures have been object of a survey among 15 general patients aged 50 years or older recruited through a Patient Organization conducted between 11-13 April 2020, who confirmed that what we propose is in line with their expectations. In particular, 91% stated that they feel able to reach a COVID-19 testing site without public transportation, 63% would be willing to read an information sheet on location regarding possible inclusion in a clinical study whilst waiting to have the test sample taken, , 75% would agree to receive a paper copy of the informed consent at home (delivered by postal mail), 86% would travel to the local center/hospital to obtain

study medication AFTER receiving notice of a positive COVID-19 test, 100% would accept to receive courier delivery of study medication at home.

7.3 Assignment to Study Groups

Randomization will be performed after the signature of the informed consent for participation and the verification of the eligibility criteria. Randomization will be performed by an instructed assistant, study coordinator, or investigator directly through the electronic data capture software (REDCAP, Vanderbilt University, v9.1.24). Additional details concerning the setting and timing of randomization are provided in the Study Procedures.

7.4 Criteria for Withdrawal/ Discontinuation of Participants

If the subject permanently discontinues treatment before Day 14 or is hospitalized, then he/she needs to have the remaining scheduled visits. Because the primary efficacy analysis of the study is based upon the intention-to treat principle, the subject will be contacted for the remaining scheduled visits. If the subject cannot be contacted telephonically, the site should collect as much follow-up information as possible, including contacting a legally acceptable representative or the treating physician by telephone or by mail to determine vital status and if a hospitalization has occurred. Vital status will be obtained by reviewing the subject's medical or public records or discharge letters from other institutions, as reported in the Patient Information.

If the subject withdraws consent from the study, this must be documented in the source document and the subject will be asked to supplement the withdrawal of consent with a signed written statement documenting refusal sent per post for all subsequent contact. If the patient does not wish to send a written statement, study withdrawal will take place immediately after giving verbal notification.

8 STUDY INTERVENTION

8.1 Identity of Investigational Product(s)

8.1.1 Experimental Intervention

Pharmacodynamic effects

Enoxaparin is a LMWH with a mean molecular weight of approximately 4,500 daltons, in which the antithrombotic and anticoagulant activities of standard heparin have been dissociated. The drug substance is the sodium salt. In the in vitro purified system, enoxaparin sodium has a high anti-Xa activity (approximately 100 IU/mg) and low anti-IIa or anti thrombin activity (approximately 28 IU/mg), with a ratio of 3.6. These anticoagulant activities are mediated through anti-thrombin III (ATIII) resulting in anti-thrombotic activities in humans. Beyond its anti-Xa/IIa activity, further antithrombotic and anti-inflammatory properties of enoxaparin have been identified in healthy subjects and

patients as well as in non-clinical models. These include ATIII-dependent inhibition of other coagulation factors like factor VIIa, induction of endogenous Tissue Factor Pathway Inhibitor (TFPI) release as well as a reduced release of von Willebrand factor (vWF) from the vascular endothelium into the blood circulation. These factors are known to contribute to the overall antithrombotic effect of enoxaparin sodium. When used as prophylactic treatment, enoxaparin sodium does not significantly affect the aPTT. When used as curative treatment, aPTT can be prolonged by 1.5-2.2 times the control time at peak activity.

Pharmacokinetic properties

The pharmacokinetic parameters of enoxaparin sodium have been studied primarily in terms of the time course of plasma anti-Xa activity and also by anti-IIa activity, at the recommended dosage ranges after single and repeated SC administration and after single IV administration. The quantitative determination of anti-Xa and anti-IIa pharmacokinetic activities was conducted by validated amidolytic methods. Absorption The absolute bioavailability of enoxaparin sodium after SC injection, based on anti-Xa activity, is close to 100%. Different doses and formulations and dosing regimens can be used. The mean maximum plasma anti-Xa activity level is observed 3 to 5 hours after SC injection and achieves approximately 0.2, 0.4, 1.0 and 1.3 anti-Xa IU/ml following single SC administration of 2,000 IU, 4,000 IU, 100 IU/kg and 150 IU/kg (20 mg, 40 mg, 1 mg/kg and 1.5 mg/kg) doses, respectively. A 3,000 IU (30 mg) IV bolus immediately followed by a 100 IU/kg (1 mg/kg) SC every 12 hours provided initial maximum anti-Xa activity level of 1.16 IU/ml (n=16) and average exposure corresponding to 88% of steady-state levels. Steady-state is achieved on the second day of treatment. After repeated SC administration of 4,000 IU (40 mg) once daily and 150 IU/kg (1.5 mg/kg) once daily regimens in healthy volunteers, the steady-state is reached on day 2 with an average exposure ratio about 15% higher than after a single dose. After repeated SC administration of the 100 IU/kg (1 mg/kg) twice daily regimen, the steady-state is reached from day 3 to 4 with mean exposure about 65% higher than after a single dose and mean maximum and trough anti-Xa activity levels of about 1.2 and 0.52 IU/ml, respectively. Injection volume and dose concentration over the range 100-200 mg/ml does not affect pharmacokinetic parameters in healthy volunteers. Enoxaparin sodium pharmacokinetics appears to be linear over the recommended dosage ranges. Intra-patient and inter-patient variability is low. Following repeated SC administration, no accumulation takes place. Plasma anti-IIa activity after SC administration is approximately ten-fold lower than anti-Xa activity. The mean maximum anti-IIa activity level is observed approximately 3 to 4 hours following SC injection and reaches 0.13 IU/ml and 0.19 IU/ml following repeated administration of 100 IU/kg (1 mg/kg) twice daily and 150 IU/kg (1.5 mg/kg) once daily, respectively.

The volume of distribution of enoxaparin sodium anti-Xa activity is about 4.3 litres and is close to the blood volume.

Enoxaparin sodium is primarily metabolized in the liver by desulfation and/or depolymerization to lower molecular weight species with much reduced biological potency.

Elimination

Enoxaparin sodium is a low clearance drug with a mean anti-Xa plasma clearance of 0.74 L/h after a 150 IU /kg (1.5 mg/kg) 6-hour IV infusion. Elimination appears monophasic with a half-life of about 5 hours after a single SC dose to about 7 hours after repeated dosing. Renal clearance of active fragments represents about 10% of the administered dose and total renal excretion of active and non-active fragments 40% of the dose. Special populations

Elderly

Based on the results of a population pharmacokinetic analysis, the enoxaparin sodium kinetic profile is not different in elderly subjects compared to younger subjects when renal function is normal. However, since renal function is known to decline with age, elderly patients may show reduced elimination of enoxaparin sodium (see sections 4.2 and 4.4). Hepatic impairment In a study conducted in patients with advanced cirrhosis treated with enoxaparin sodium 4,000 IU (40 mg) once daily, a decrease in maximum anti-Xa activity was associated with an increase in the severity of hepatic impairment (assessed by Child-Pugh categories). This decrease was mainly attributed to a decrease in ATIII level secondary to a reduced synthesis of ATIII in patients with hepatic impairment.

Renal impairment

A linear relationship between anti-Xa plasma clearance and creatinine clearance at steady-state has been observed, which indicates decreased clearance of enoxaparin sodium in patients with reduced renal function. Anti-Xa exposure represented by AUC, at steady-state, is marginally increased in mild (creatinine clearance 50-80 ml/min) and moderate (creatinine clearance 30-50 ml/min) renal impairment after repeated SC 4,000 IU (40 mg) once daily doses. In patients with severe renal impairment (creatinine clearance <30 ml/min), the AUC at steady state is significantly increased on average by 65% after repeated SC 4,000 IU (40 mg) once daily doses (see sections 4.2 and 4.4).

Haemodialysis

Enoxaparin sodium pharmacokinetics appeared similar than control population, after a single 25 IU, 50 IU or 100 IU/kg (0.25, 0.50 or 1.0 mg/kg) IV dose however, AUC was two-fold higher than control.

Weight

After repeated SC 150 IU/kg (1.5 mg/kg) once daily dosing, mean AUC of anti-Xa activity is marginally higher at steady state in obese healthy volunteers (BMI 30-48 kg/m2) compared to non-obese control subjects, while maximum plasma anti-Xa activity level is not increased. There is a lower weight-adjusted clearance in obese subjects with SC dosing. When non-weight adjusted dosing was administered, it was found after a single-SC 4,000 IU (40 mg) dose, that anti-Xa exposure is 52% higher in low-weight women (<45 kg) and 27% higher in low-weight men (<57 kg) when compared to normal weight control subjects (see section 4.4).

Pharmacokinetic interactions

No pharmacokinetic interactions were observed between enoxaparin sodium and thrombolytics when administered concomitantly.

List of excipients

Water for Injections.

Shelf life

2 years.

Nature and contents of container

Clexane® Forte Syringes 12,000 IU (120 mg)/0.8 mL and 15,000 IU (150mg)/1 mL: Solution for injection in graduated pre-filled syringes (type I glass) fitted with rubber stopper (chlorobutyl and bromobuytl) and injection needle (with automatic safety system ERISTM or PREVENTIS™ or without an automatic safety system). Supplied in packs of 2, 5, 6, 10, 20, 30, 50 pre-filled syringes and in multi-packs of 3 x 10 pre-filled syringes. Not all pack sizes may be marketed.

Special precautions for disposal and other handling

Pre-filled syringes are ready for immediate use. For method of administration see section 4.2. Use only clear, colourless to yellowish solutions. Pre-filled syringes are supplied with or without an automatic safety system. The instructions for use are presented in the package leaflet. Each syringe is for single use only. Used syringes will be discarded into regular trash without trying to recap the already automatically retracted needle. Any unused medicinal product (enoxaparin prefilled injections) should be returned back to the study center at a later timepoint (>5 days) due to potential contamination.

8.1.2 Control Intervention

No treatment.

8.1.3 Packaging, Labelling and Supply (Re-Supply)

The Sponsor will label the study medication centrally and ship an initial amount of the study medication to the participating centers, which should be sufficient for the enrolment of the first 80 patients per center and will consist of 10- and 2-syrynge packages. The shipment will be performed in accordance with storage requirements (see below). Upon receipt of the study treatment supplies, an inventory must be performed and a drug receipt log filled out and signed by the person accepting the shipment.

It is important that the designated study staff counts and verifies that the shipment contains all the items noted in the shipment inventory. Any damaged or unusable study drug in a given shipment will be documented in the study files and the sponsor will be notified by the study-site personnel within 24 hours after being aware of the event.

The investigator is responsible for ensuring that all study drug received at the site is inventoried and accounted for throughout the study. The dispensing of study drug to the subject must be documented on the drug accountability form, signed and dated by the study team.

8.1.4 Storage Conditions

Do not store above 25°C. Do not freeze.

8.2 Administration of experimental and control interventions

8.2.1 Experimental Intervention

Enoxaparin (Clexane®) will be given at the recommended dose of 4,000 IU antiXa activity (40 mg)/0.4 ml once daily by SC injection for 14 days.

The first dose of enoxaparin will be administered on the day of randomization. The subsequent doses of enoxaparin will be administered or self-administered at home.

Instructions on how to administer enoxaparin will be provided during the screening/baseline visit. Additional details are reported in the Study Procedures.

Pharmaceutical form

Solution for injection in pre-filled syringes. Clear, colourless to yellowish solution, pH-value 5.5-7.5.

Method of administration

Enoxaparin should not be administered by the intramuscular route. For the prophylaxis of venous thromboembolic disease following surgery, treatment of DVT and PE, treatment of unstable angina and NSTEMI, enoxaparin sodium should be administered by SC injection.

The pre-filled disposable syringe is ready for immediate use.

SC injection technique: Injection should be made preferably when the patient is lying down. Enoxaparin sodium is administered by deep SC injection. Do not expel the air bubble from the syringe before the injection to avoid the loss of drug when using pre-filled syringes. The administration should be alternated between the left and right anterolateral or posterolateral abdominal wall, and upper legs.

8.2.2 Control Intervention

Not applicable.

8.3 *Dose Modifications*

In accordance with the label of enoxaparin, no dose modification is requested.

In case of hospitalization (component of the primary efficacy outcome) or bleeding (secondary outcome) or any other adverse event that may occur, the dose can be

modified based on what decided by the treating physician, as per standard clinical practice. This will not influence the primary efficacy outcome analysis, which will be conducted on a intention-to-treat basis.

8.4 Compliance with Study Intervention

The study team will assess and track participant compliance during the scheduled phone follow-up visits (Day 3, 7 and Day 14) and according with a pre-defined questionnaire evaluating the use of the study medication and potentially related adverse events.

8.5 Data Collection and Follow-up for Withdrawn Participants

If a patient withdraws from the study, data until the date of withdrawal will be used for analysis.

8.6 Trial Specific Preventive Measures

If a subject has a serious bleeding event during study drug treatment, the following routine measures should be considered:

- Delay the next study drug administration, or discontinue treatment if indicated. Temporary cessation of study drug may allow control of bleeding.

Consider the usual treatment measures for bleeding events, including fluid replacement and hemodynamic support, blood transfusion, and fresh frozen plasma, if physical examination and laboratory testing suggest benefit could be obtained.

In case of life-threatening bleeding occurring during enoxaparin administration, protamine may be used for reversal, as per standard management, although it is known that it only partially reverses the anti-Xa activity and there are very limited data on clinical effectiveness.
If deemed necessary, enoxaparin reversal can be determined based on the following table (Lovenox [package insert]. Sanofi-Aventis U.S. LLC; Bridgewater, NJ. October 2013):

≤ 8 hours since heparin dose: 1 mg protamine for every 1 mg enoxaparin;
> 8 hours since heparin dose: 0.5 mg protamine for every 1 mg enoxaparin;
> 12 to 24 hours since heparin dose: depending on dose received and renal function, protamine reversal may not be necessary due to enoxaparin metabolism.

8.7 Concomitant Interventions

All concomitant and/or rescue treatment(s) have to be recorded in the eCRF.

The use of other anticoagulant agents is not recommended during the study period, as it may significantly increase the risk of bleeding. If another anticoagulant agent is deemed necessary (i.e. for a new diagnosis of atrial fibrillation), the switching from enoxaparin to the new anticoagulant agent will be performed according with standard procedures.

Paracetamol should be considered the drug of choice to treat fever or pain. The use of ibuprofen (or other NSAIDs) is not forbidden, although its administration should be discussed with the treating or study physician. Similarly, the introduction of an antiplatelet therapy should be discussed with the treating physicians and principally be avoided in the absence of a major (e.g. cardiologic) indication.

8.8 Study Drug Accountability

Investigational product supplies, which will be provided to the Principal Investigator of each site, must be kept in a secure, limited access storage area under the recommended storage conditions. Lot number and expiry date should be listed. The investigator will maintain accurate and adequate records including dates, lot number, quantities received, usage. In addition, a study diary will be handed to patients in the treatment group and patients will be encouraged to document forgotten or missed doses (as described in 7.2). A missed injection can be made up for up to 6 hours, not used study products should be returned to the site. The used study product will not be collected, but disposed through the regular household waste.

8.9 Return or Destruction of Study Drug

At the completion of the study, there will be a final reconciliation of drug shipped, drug consumed, and drug remaining. This reconciliation will be logged on the drug accountability form, signed and dated. Any discrepancies noted will be investigated, resolved, and documented. Drug destroyed on site will be documented in the study files.

9 STUDY ASSESSMENTS

9.1 Study Flow Chart/Table of Study Procedures and Assessments

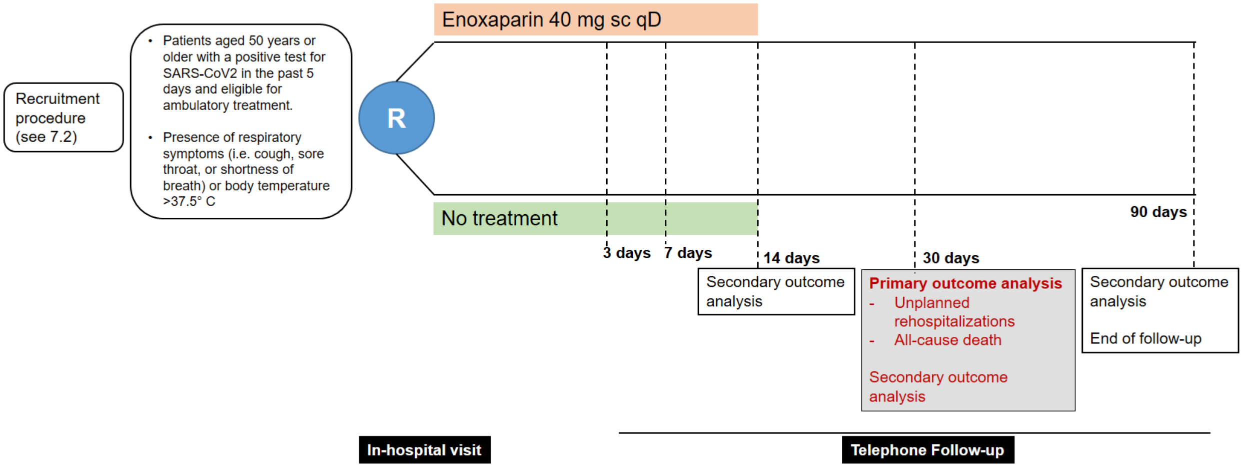

9.2 Assessments of Outcomes

9.2.1 Assessment of Primary Outcome

The primary outcome (any hospitalization or all-cause death) will be assessed by direct telephone contact with the patient, with the contact person, or with the treating physicians (Day 3, 7, 14, 30). Due to the unquestionable nature of the primary efficacy outcome, no adjudication of the events is deemed necessary.

The investigators will be allowed by the patient to obtain clinical information from discharge letters in case of hospitalization or medical treatment. All the information collected telephonically based on a predefined questionnaire and conversation notes will be documented (patient questionnaire, visits 2-6); the document will be regarded as source document. Additional source documents will be represented by admission/discharge letters in the case the patient will undergo ambulatory visits or will be admitted to the hospital (documentation of outcomes). Primary and secondary endpoints must be documented by written reports of the treating physicians. After termination of the phone call, all information will be transferred into the eCRF. Telephone contact will occur at predefined timepoints: patients (or the first contact person) will be contacted on the day of the planned contact. In case the patient (or the first contact person) cannot be contacted, a second phone call will take place two hours later. In case of no answer, the second contact person will be immediately contacted and the same procedure will take place. If the patient (or contact persons) cannot be reached by phone, the treating physicians will be alerted due to safety reasons.

9.2.2 Assessment of Secondary Outcomes

9.2.3 Cardiovascular and thromboembolic complications (i.e. venous thromboembolism, myocardial infarction or stroke) will be evaluated at day 3, 7, 14, 30 and 90 equivalently to the primary outcome (9.2.1). Assessment of Safety Outcomes

Major bleeding and non-major clinically relevant bleeding will be evaluated at day 3, 7, 14, 30, 90 by asking specific questions on the phone (cf. question list file “CRF Variables List, page “Phone Visit”, “Primary outcome”, “Secondary outcome”). If the patient cannot be reached by phone or if clarification is needed, the treating physician of the patient will be contacted.

9.2.3.1 Serious Adverse Events

Recording of serious adverse event is described in section 10.

9.2.3.2 Laboratory Parameters

No information on specify laboratory parameters will be routinely assessed during the course of the study as part of the study outcomes: the follow-up visits will take place telephonically.

9.2.3.1 Vital Signs

No information on vital signs will be routinely assessed during the course of the study as part of the study outcomes: all the follow-up visits will take place telephonically.

9.2.4 Assessments in Participants Who Prematurely Stop the Study

No additional investigation is planned in participants who voluntarily stop the study for non-medical reasons.

9.3 Procedures at each visit

9.3.1 Pre-screening period (Day -5 to Day 1)

Pre-screening (Day -5 to 1) will take place according with the scenarios summarized in Section 7.2 as the process of screening may vary according with individual capacities and setting of initial SARS-CoV2 testing.
Procedures for pre-screening, irrespective of the scenario:

- Signature of PIC1 (if applicable)
- Discussion with the patient on study participation (pre-screen survey)
- Referral to the closest study center.

9.3.2 Screening and baseline Visit (Day 1)

Procedures at baseline visit, irrespective of the scenario:

- Signature of PIC2
- Collection of demographic and baseline characteristics, including comorbidities and comedications
- Vital signs assessment: respiratory rate, heart rate, systolic blood pressure, diastolic blood pressure, body temperature, oxygen saturation, height, weight, body mass index
- If not available, additional blood test analysis with point of care or standard assays.
- If the subject signed PIC2 and meets all of the inclusion and none of the exclusion criteria, he or she is eligible to be randomly assigned to receive enoxaparin or no therapy (randomization)
- Allocation to study treatment
- If applicable, patients will be instructed on how to administer the study medication via personal communication, remote instructions, and/or video material.
- First injection of enoxaparin

For bleeding events, subjects and family members as appropriate, will be instructed:

- To seek medical attention if they develop bleeding
- To contact the investigative site staff or study investigator before the next dose of study medication is due
- To inform treating health care providers about study participation

Subjects and family members, as appropriate will also be instructed:

- About the subject’s risk of DVT and PE
- About the signs and symptoms of DVT and PE
- To seek medical attention if they develop any of these signs or symptoms

- To contact the investigative site staff or study investigator as soon as symptoms develop and before the next dose of study medication is due
- To inform treating health care providers about hospitalization or death.

The subject's family should be instructed to have a low threshold to contact the site (a patient card is given).

9.3.3 Follow-up visits, including Final visit

Follow-up visits: phone contact and assessment of the primary/secondary study outcomes (Day 3, 7, 14, 30, and 90), suspected serious adverse events (Day 3, 7, 14, 30, and 90), vital status (Day 3, 7, 14, 30, and 90), drug compliance (Day 3, 7 and 14). Information on symptoms potentially pointing to a study outcome or SAE will be adequately collected during the follow-up visit and the patient may be instructed to seek in-person medical assistance if deemed necessary by the study personnel, i.e. in the case of a suspected thromboembolic event (DVT, PE, myocardial infarction stroke), bleeding with characteristics of severity (i.e. occurring at critical sites), substantial worsening of the respiratory symptoms compared with baseline, onset of new symptoms, SAE.

10 SAFETY

The Sponsor's SOPs will provide more detail on safety reporting.

During the entire duration of the study, all serious adverse events except the study outcomes as noted above will be collected and entered into the serious adverse event page of the eCRF. Study duration encompasses the time from when the participant signs the informed consent until the last protocol-specific procedure has been completed (phone call at day 90).

10.1 Definitions

Serious Adverse Event

An SAE is defined as any untoward medical occurrence that at any dose results in

• results in death,

• is life-threatening,

• requires participant hospitalization or prolongation of current hospitalization,

• results in persistent or significant disability/incapacity, or

• is a congenital anomaly/birth defect,

• any important medical event and any event which, though not included in the above, may jeopardise the participant or may require intervention to prevent one of the outcomes listed above.

Any other medically important condition that may be not immediately life-threatening or results in death or hospitalization but may jeopardize the participant or may require

intervention to prevent one of the outcomes listed above should also usually (i.e. based on medical and scientific judgment) be considered serious.

Unexpected Adverse Drug Reaction

An "unexpected" adverse drug reaction is an adverse reaction, the nature or severity of which is not consistent with the applicable product information.

Suspected unexpected serious adverse reaction (SUSAR)

A serious adverse reaction, the nature or severity of which is suspected to be not consistent with the applicable product information.

Safety Signals

All suspected new risks and relevant new aspects of known adverse reactions that require safety-related measures.

10.2 Recording of Serious Adverse Events

Clinical investigators and ultimately the Principal Investigator (PI) have the primary responsibility for SAE identification, documentation, grading, and assignment of attribution to the investigational agent/intervention.

Information on all SAEs will be collected during phone follow-up. Standard questionnaire (Patient Questionnaire, Visits 2-6) will include key questions for SAE screening, including the occurrence of the primary efficacy outcome (hospitalization, death), bleeding at potentially critical sites, or symptoms that may reflect an underlying and potentially severe condition of new onset. In the latter case, the severity of symptoms will be compared with the status at baseline.

All SAEs will be fully documented in the appropriate eCRF. For each SAE, the investigator will provide the onset, duration, intensity, treatment required, outcome and action taken with the investigational product.

The investigator assesses the causal relationship of each SAE according to the SAE reporting form.

10.3 Assessment of Serious Adverse Events

An unexpected SAE refers to any AE, the nature or severity of which is not consistent with the applicable product information.

The investigator will promptly review SAEs to determine if the SAE meets the criteria for a SUSAR.

The assessment by the investigator with regard to the study drug relation is done according to the following definitions:

Unrelated	• The event started in no temporal relationship to medicinal product applied and<br>• The event can be definitely explained by underlying diseases or other situations.
Related	• The event started in a plausible temporal relationship to medicinal product applied and

	• The event cannot be definitely explained by underlying diseases or other situations.

10.4 Reporting of Serious Adverse Events

The investigator is responsible for reporting any SAEs to the Sponsor immediately, i.e. within 24 hours using the following email address: ovidstudie@usz.ch

The Investigator is responsible for SAE reporting to the CEC according to the following details:

- Reporting to CEC any SAE which resulted in death:

  - **without delay**, and no later than **7 calendar days**.

- Reporting to CEC of fatal SAEs if evaluated as "suspected", "unexpected" and "drug related" (SUSAR)

  - **without delay** and no later than **7 calendar days** following awareness that event meets criteria for an SUSAR.

- Reporting to CEC of non-fatal SAEs if evaluated as "suspected", "unexpected" and "drug related" (SUSAR):

- **promptly** and no later than **15 calendar days** following awareness that event meets criteria for a SUSAR.

The Sponsor is responsible for SAE reporting to Swissmedic according to the following details:

• Compliance with the regulatory requirements of Swissmedic regarding prompt reporting of unexpected SAEs for which a causal relationship with the study drug cannot be ruled out.

• Reporting to Swissmedic of fatal SAEs if evaluated as "suspected", "unexpected" and "drug related" (SUSAR):

- without delay and no later than 7 calendar days following awareness that event meets criteria for a SUSAR;

• Reporting to Swissmedic of non-fatal SAEs if evaluated as "suspected", "unexpected" and "drug related" (SUSARs):

- promptly and no later than 15 calendar days following awareness that event meets criteria for a SUSAR.

All other SAEs will be summed up in the **annual safety report (ASR)**, containing:

- A summary of the safety profile of the drug studied as well as the safety issues that have arisen;
- A listing of all SUSARs that have occurred in Switzerland
- The accompanying letter provided with the Annual Safety Report should contain a short summary of the status of the clinical trial in Switzerland (number of

centers open/closed, number of patients recruited/recruitment closed, and number of SADR/SUSAR.

The Sponsor is responsible for the reporting of unexpected ADRs to Swissmedic according to the normal pharmacovigilance practice.

Reporting of Safety Signals
All suspected new risks and relevant new aspects of known adverse reactions that require safety-related measures, i.e. so-called safety signals, must be reported to the Sponsor-Investigator within 24 hours. The Sponsor-Investigator must report the safety signals within 7 days to the local Ethics Committee (local event via local Investigator).
The Sponsor-Investigator must immediately inform all participating Investigators about all safety signals. The other in the trial involved Ethics Committees will be informed about safety signals in Switzerland via the Sponsor-Investigator.

10.1 Data Safety Monitoring Board (DSMB)

The Sponsor is responsible to prepare a DSMB Charter, which will be submitted to the KEK and Swissmedic in due time and before the enrolment of the first patient.
It is the role of the DSMB to advise the Sponsor regarding the continuing safety of current participants and those yet to be recruited, as well as the continuing integrity, validity and scientific merit of the trial. A fundamental consideration is the safety of those who would be at potential risk due to their participation in the trial. The DSMB must be particularly alert to the risks inherent to anticoagulant therapy.
In addition, the conduct of the study is subject to review in the context of its capability to ultimately address the scientific questions of interest, including recruitment rate, ineligibility, non-compliance, protocol violations, dropouts, completeness and timeliness of data.
The DSMB may advise the Sponsor and Principal Investigators to modify/improve specific aspects of the study conduct. The DSMB has a more circumspect role in recommending changes to the study protocol after having discussed these concerns with the Sponsor and Principal Investigators.
Members of the DSMB have relevant expertise and experience in clinical trials and are aware of the responsibilities inherent in the operation of the DSMB. The DSMB includes a biostatistician knowledgeable in statistical methods used in clinical trials.

10.2 Follow up of Serious Adverse Events

All serious adverse events that have not resolved by the end of the study, or that have not resolved upon discontinuation of the subject's participation in the study, will be followed until any of the following occurs:

- The event resolves,
- The event stabilizes,
- The event returns to baseline, if a baseline value/status is available,
- The event can be attributed to agents other than the study drug or to factors unrelated to study conduct,

- It becomes unlikely that any additional information can be obtained (subject or health care practitioner refusal to provide additional information, lost to follow-up after demonstration of due diligence with follow-up efforts).

11 STATISTICAL METHODS

11.1 Hypothesis

Two treatment arms are to be compared, experimental arm with enoxaparin versus control arm without any treatment. Randomization will be 1:1. Objective is to demonstrate superiority of experimental treatment (enoxaparin). Primary outcome of the study is any hospitalization or any-cause death within 30 days of enrolment.

Sample size is fixed, one interim analysis is planned at time when the outcomes of 50% of the patients have been observed.

11.2 Determination of Sample Size

We obtained official data on fatality and hospitalization rates observed in the Swiss population until 31.03.2020: a total of 12.372 patients aged 50 years or older tested positive for SARS-CoV-2, of whom 645 (5.2%) died and 2.350 (19.0%) were hospitalized irrespective of whether this consisted of a primary hospitalization (after evaluation at the emergency department) or a secondary hospitalization for clinical deterioration after initial ambulatory treatment. Assuming that two thirds of deaths and of any hospitalizations occurred in ambulatory patients, we estimated that the primary efficacy outcome rate would occur in 15% (any hospitalization 11%, case fatality rate 4%). As we anticipate that the a substantial number of the primary endpoint is due to venous or arterial thromboembolic complications, which would be prevented by the use of prophylactic-dose enoxaparin, we estimated that enoxaparin will decrease the primary efficacy outcome to 9% (RR 0.6).
The following studies were also considered for estimating the benefit of enoxaparin use in medical patients and sample size calculation:
- Prophylactic treatment with 40 mg per day of enoxaparin subcutaneously (vs. placebo) safely reduced the risk of venous thromboembolism detected by bilateral venography or duplex ultrasonography in patients with acute medical illnesses (5.5 percent [16 of 291 patients]) in the group that received 40 mg of enoxaparin vs. 14.9 percent [43 of 288 patients] in the group that received placebo) (relative risk, 0.37; 97.6 percent confidence interval, 0.22 to 0.63) (31).
- Acutely ill medical patients on enoxaparin plus elastic stockings with graduated compression had a 14-day rate of sudden death or pulmonary embolism of 0.5% as compared with 0.7% in those with elastic stockings with graduated compression alone (RR 0.7; 95%CI 0.4-1.3) (65).
- In EXCLAIM, long-term (28-day ±4) enoxaparin 40 mg/d vs. 10-day (±4) enoxaparin 40 mg/d reduced proximal VTE (2.4% vs 3.8%) (66).

- In a meta-analysis of phase III trials, extended thromboprophylaxis in medical patients reduced symptomatic VTE or VTE-related death compared with standard of care (0.8% versus 1.2%; risk ratio [RR]: 0.61, 95%CI 0.44-0.83) (49).
- Anticoagulant therapy, mainly with LMWH, appeared to be associated with better prognosis in severe COVID-19 patients meeting SIC criteria or with markedly elevated D-dimer (4).

The sample size calculation is based on the parameters α= 0.05 (2-sided), power = $1-\beta$ = 0.8, event rate in experimental group, $pexp$= 0.09 and event rate in control group, $pcon$= 0.15. The resulting total sample size is 920. To account for potential drop-outs, the total sample size was fixed to 1000. This will be the maximum sample size, no increase in sample size is planned. Results will be reported in terms of risk ratios (RR) between experimental and control group, i.e. we anticipate that the estimated RR will be < 1.

11.3 Statistical Criteria of Termination of Trial

See details reported in the Interim Analysis (11.4.4).

11.4 Planned Analyses

11.4.1 Datasets to be Analysed, Analysis Populations

A detailed statistical analysis plan will be written up upon ethics approval.

11.4.2 Primary Analysis

The primary efficacy outcome analysis will be conducted in the intention-to-treat (ITT) population, consisting of all randomized subjects who signed a valid informed consent.
Descriptive statistics of the patient characteristics at baseline will include mean and standard deviation for continuous variables, median and interquartile range for the ordinal or non-normal variables, as well as numbers and percentages of total for the categorical variables. For the primary outcome, the relative risk will be calculated for the experimental group as compared to control group, with 95% confidence interval. Refined analyses include the stratification variables in order to reduce unexplained heterogeneity. For that, the Mantel Haenszel method as well as multiple logistic regression models will be used. A more detailed statistical analysis plan for interim analysis and final analysis will be written up while patients are being recruited. All analyses will be conducted with R (R Core Team 2019). RMarkdown will be used for dynamic reporting. R-packages used for sample size determination and study design: IAbin 1.0, gsDesign 3.0.1, and TrialSize 1.3.The corresponding reporting guideline for randomized superiority trials is CONSORT guideline.
Heterogeneity analysis to study treatment effects of enoxaparin (versus no treatment) will be conducted in specific subgroups of patients categorized by sex, age-groups, renal function, and concomitant antiplatelet therapy.

11.4.3 Secondary Analyses

Groups will be compared for all the secondary outcomes. Results will be reported as relative risk and they will be calculated for the experimental group as compared to control group, with 95% confidence interval.

11.4.4 Interim Analyses

A single interim analysis is planned. The aim of the interim analysis is to stop the trial early for efficacy (superiority) or futility. A group-sequential approach will be used, based on stopping boundaries with O'Brian-Fleming design (OBF). OBF is accepted by regulators and ICH-E9 guidelines.

```
##   Analysis                Value Efficacy Futility
##  IA 1: 50%                    Z   2.7965  -2.7965
##     N: 499        p (1-sided)   0.0026   0.0026
##                     RR at bound   0.4876   2.0510
##                 P(Cross) if RR=1   0.0026   0.0026
##               P(Cross) if RR=0.6   0.2096   0.0000
##      Final                    Z   1.9774  -1.9774
##     N: 997        p (1-sided)   0.0240   0.0240
##                     RR at bound   0.6983   1.4321
##                 P(Cross) if RR=1   0.0250   0.0250
##               P(Cross) if RR=0.6   0.8000   0.0000
```

Design specifications: a symmetric 2-sided group sequential design with 2 analyses and a total sample size of 997 patients will be used, power will be 80%, 1-sided significance level α will be 0.025 (type I error). Bounds were derived using OBF boundary. The following plot shows an illustration of the specification.

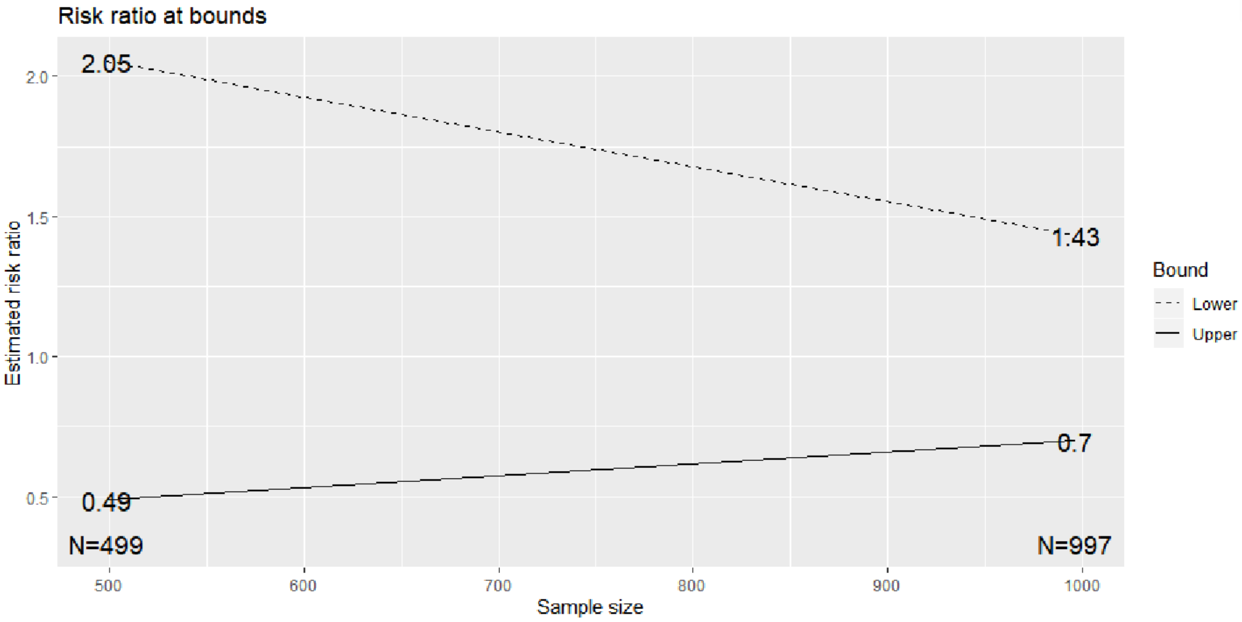

Figure 1: Illustration of the risk ratio and sample size at interim and final analysis.
The bounds correspond to different directions in which the treatment effect could go. Stopping the trial early because of safety concerns and futility needs no adjustment of the overall significance level α.

11.4.5 Safety Analysis

The analysis of safety outcomes will be conducted in the safety population, including patients who received at least one dose of study drug (enoxaparin) or who were alive 12 hours after randomization.

12 ELIGIBILITY OF THE PROJECT SITE(S)

The OVID study will be conducted as a national research initiative involving all the five university hospitals and two large cantonal hospitals in Ticino under the coordination of a local COVID Commitee.

Requirements OVID centers:

1) At least one patient room is required for enrolment, ideally located outside main hospital facilities to avoid transmission of COVID to staff and other patients
2) Ability to locally perform rapid blood cell count (hemoglobin, platelet count) and creatinine tests as some COVID-19 outpatients may have no recent (<3 months) results of blood tests mandatory for enrolment. If point of care tests are used, these should ideally be performed outside the patient rooms.
3) Parking lot nearby the patient rooms because COVID patients may not use public transport.
4) Signed contract with University Hospital Zurich (recruitment and study procedures must be in accordance with GCP standards and performed according with at least one of the aforementioned scenarios).

Each participating center has already received a contract examined by Unitectra (Clinical Research Agreement), which includes data on financing, results, data and liability, etc…

13 DATA QUALITY ASSURANCE AND CONTROL

The sponsor investigator is responsible for the implementation and maintenance of a quality management system, including the performance of quality controls in the form of monitoring and, if necessary, quality assurance audits.

The sponsor investigator provides study-specific SOPs and WIs to the participating centres.

The investigator is responsible for ensuring that all persons involved in the trial are adequately trained for their tasks. This ensures that the test procedures are carried out in a standardized manner and that the applicable guidelines and laws are observed

13.1 DATA HANDLING AND RECORD KEEPING

The study will strictly follow the protocol. If any changes become necessary, they must be laid down in an amendment to the protocol. All amendments of the protocol must be signed by the Sponsor-Investigator and will be submitted to CEC and Swissmedic.

13.1.1 Case Report Forms

The investigators will use electronic case report forms (eCRF), one for each enrolled study participant, to be filled in with all relevant data pertaining to the participant during the study. All participants who signed the informed consent (PIC2) have to be documented on a screening log. The investigator will document the participation of each study participant on the enrolment Log.

For data and query management, monitoring, reporting and coding an internet-based secure electronic data capture system RedCap, which is hosted by the Clinical Trials Centre (CTC) Zurich will be used for this study. It is the responsibility of the investigator to assure that all data in the course of the study will be entered completely and correctly in the respective data base. Corrections in the eCRF may only be done by the investigator or by other authorised persons. In case of corrections the original data entries will be archived in the system and can be made visible. For all data entries and corrections date, time of day and person who is performing the entries will be generated automatically.

eCRFs must be kept current to reflect participant status at each phase during the course of study. Participants must not to be identified in the eCRF by name. Appropriate coded identification (e.g. Participant Number) must be used.

It will be assured that any authorised person, who may perform data entries and changes in the eCRF, can be identified. A list with signatures and initials of all authorised persons will be filed in the study site file and the trial master file, respectively.

The investigators assure to perform a complete and accurate documentation of the participant data in the eCRF. All data entered into the eCRF with exception of (for which data the eCRF will be source data to be specified for each study) must also be available in the individual participant file either as print-outs or as notes taken by either the investigator or another responsible person assigned by the investigator.

13.1.2 Specification of Source Documents

The following documents are considered source data, including but not limited to:

- eCRF (demographic and baseline characteristics, vital signs, comedications, laboratory parameters, SAE, study outcomes),
- Nurse records, records of clinical coordinators, and
- Medical records from other department(s), or other hospital(s), or discharge letters and correspondence with other departments/hospitals.

Source data must be available at the site to document the existence of the study participants and substantiate the integrity of study data collected. Source data must include the original documents relating to the study, as well as the medical treatment and medical history of the participant.

The following information (at least but not limited to) should be included in the source documents:

• Demographic data (age, sex)
• Inclusion and Exclusion Criteria details
• Participation in study and signed and dated Informed Consent Forms
• Visit dates
• Medical history and physical examination details
• Key efficacy and safety data (as specified in the protocol)
• AEs and concomitant medication
• Results of relevant examinations
• Laboratory printouts
• Dispensing and return of study drugs
• Reason for premature discontinuation
• Assignment to treatment group

The data collected during the phone calls (day 3, 7, 14, 30 and 90) will be entered directly in the eCRF. Additionally, the site reports from the visits (in-hospital visit, as well as phone visits) will be imported into the electronic patient chart of the study centers.

13.1.3 Record Keeping / Archiving

All study data must be archived for a minimum of 10 years after study termination or premature termination of the clinical trial.
Any patient files must be archived for the longest possible period of time according to the feasibility of the investigational site, e.g. hospital, institution or private practice

13.2 Data Management

13.2.1 Data Management System

For the present clinical study, the electronic data capture (EDC) software REDCap (www.project---redcap.org) will be used for data processing and management. REDCap was developed by an informatics core at Vanderbilt University in 2004, with ongoing support from US National Center for Research Resources (NCRR) and US National Institute of Health (NIH), grants NIH/NCATS UL1 TR000445. REDCap was specifically developed around HIPAA security guidelines and is GCP-compliant and fulfills the Swiss regulatory requirements regarding the collection of patient data in clinical trials or non-interventional studies and patient registries and the Swiss/EU data protections laws.

Operating requirements include a Linux, UNIX, Windows or Mac interface. The system requires a SMTP e-mail server, is accessed via PHP web-based Front End (e.g. Microsoft IIS or Apache) and runs on a MySQL database server, hosted by the Clinical Trials Center of the University Hospital Zurich, which holds a REDCap End-User License Agreement for this EDC system.

Data collection occurs via electronic Case Report Forms (eCRFs), which are generated via study-specific data dictionary defined in an iterative self-documenting process by members of the research team with planning support from the Data Management Department of the Clinical Trials Center, University Hospital Zurich, Switzerland (CTU Zurich). The iterative development and testing process results in a well-planned data collection strategy in keeping with the outcome parameters and procedures defined in the

study protocol.

Ongoing maintenance and use of this software is contractually agreed upon between the study sponsor and the Data Management Department of the CTU Zurich.

13.2.2 Data Security, Access and Back-up

Appropriate coded identification (e.g. pseudonymisation) is used in order to enter subject data into the database. All data entered into eCRFs is transferred to a mySQL database using encryption post filtering and sanitization to various relational database tables.

The server hosting the EDC system and the database is kept in an off-site locked server-room. Only system administrators have direct access to the server and back-up tapes.

A role-based user concept with personal login and passwords (e.g. for site investigator, statistician, monitor, administrator etc.) regulates permission for each user to access the system and database when required.

Within each project, there are role- and user-based settings to control access to various functionality and modules, such as being able to export data, to enter data, export reports and view the logging records. Another feature called Data Access Groups, can be implemented to help segregate users so that the data they enter is only accessible by someone in their group, especially useful for multi-centric studies where the data entered by one institution should not be accessible or viewable by others within the same project. A current list with signatures and names of all authorized study personnel with access to the study records will be filed in the study site file and the trial master file, respectively.

A built-in data logging tool (audit trail) ensures that any changes to the project or user activity (date and time stamp and user log), including contextual information (e.g. the project record being accessed), are continuously tracked in real-time and accessible online or via downloadable audit table.

A multi-level back-up system is in place. Whole system internal back-ups including the database are run several times per day and an additional external back-up onto tape once a day. The back-up tapes are stored in a secure place in a separate building.

13.2.3 Analysis and Archiving

eCRFs are kept current to reflect subject status at each phase during the course of the study. For ad interim (if applicable) and final analyses, data files are extracted from the database in CSV (case-delimited) format, typically supported by Microsoft Excel, SAS, Stata, R, or SPSS software systems. Direct import into these statistical packages is advised for best data analyses. This study foresees the use of R for statistical analysis of study outcome.

The study database will be securely stored by CTU Zürich for at least 15 years (after the regular end or a premature termination of the respective study).

13.2.4 Electronic and Central Data Validation

The EDC system supports data checks completeness and plausibility. Furthermore, selected data points are cross-checked for plausibility with previously entered data for that participant. Additional central data validity checks against pre-determined parameters are run either automatically or ad hoc, to detect inconsistencies and identify missing data for source data verification.

13.3 Monitoring

Monitoring prior to the start and during the course of the study will help to follow up the progress of the clinical study, to assure utmost accuracy of the data and to detect possible errors at an early time point. The Sponsor-Investigator organises professional independent monitoring for the study and will collaborate with the Clinical Trials Center (CTC) of the University Hospital Zurich. According to the CTC's Monitoring SOP the extent and nature of monitoring activities based on the objective and design of the study will be defined in a study specific Monitoring Plan. During the COVID-19 pandemic monitoring will be performed by remote techniques as defined in the monitoring plan.

See the attached Monitoring Plan.

13.4 Audits and Inspections

A quality assurance audit/inspection of this study may be conducted by the competent authority or CEC, respectively. The quality assurance auditor/inspector will have access to all medical records, the investigator's study related files and correspondence, and the informed consent documentation that is relevant to this clinical study.

The investigator will allow the persons being responsible for the audit or the inspection to have access to the source data/documents and to answer any questions arising. All involved parties will keep the patient data strictly confidential.

13.5 Confidentiality, Data Protection

Direct access to source documents will be permitted for purposes of monitoring, audits and inspections.

13.6 Storage of Biological Material and Related Health Data

Not applicable.

14 PUBLICATION AND DISSEMINATION POLICY

By signing the clinical trial protocol, the investigator agrees on the use of the results of this clinical trial for publication and information for medical and industrial professionals. The findings of this clinical trial including the interim analysis will be published in a scientific journal or presented at a scientific meeting and may be used for pooled analyses with similar trials. Publication of clinical trial results requires mutual agreement between the investigators and the sponsor. Any publication of the clinical trial data by the sponsor or investigators will be wholly consistent with the integrated report in accordance with the ethical principles of the Declaration of Helsinki.
All publications will follow the Uniform Requirements for Manuscripts Submitted to Biomedical Journals (www.icmje.org, October 2008).

15 FUNDING AND SUPPORT

15.1 Funding

This investigator-initiated study will be funded by the Clinic of Angiology, USZ and the Clinic of Cardiology, Inselspital. Various applications for public funding have been conducted, including University Zurich, Innovation Pool USZ, Swiss Red Cross froundation, Johanna Dürmüller-Bol foundation. Additional public funding will be requested as needed.

15.2 Other Support

In case of involvement of centers from other countries, potential co-sponsors will apply for separate national public fundings.

16 INSURANCE

Insurance is covered by "Versicherung für klinische Versuche und nichtklinische Versuche" by Zürich Versicherungs-Gesellschaft AG (Policy no: 14.970.888).

Any damage developed in relation to study participation is covered by this insurance. So as not to forfeit their insurance cover, the participants themselves must strictly follow the instructions of the study personnel. Participants must not be involved in any other medical treatment without permission of the principal investigator (emergency excluded). Medical emergency treatment must be reported immediately to the investigator. The investigator must also be informed instantly, in the event of health problems or other damages during or after the course of study treatment.

The investigator will allow delegates of the insurance company to have access to the source data/documents as necessary to clarify a case of damage related to study participation. All involved parties will keep the patient data strictly confidential.

A copy of the insurance certificate will be placed in the Investigator's Site File.

17 REFERENCES

1. Corriere dell Sera - Data Analysis. «The real death toll for Covid-19 is at least 4 times the official numbers». Available at: https://www.corriere.it/politica/20_marzo_26/the-real-death-toll-for-covid-19-is-at-least-4-times-the-official-numbers-b5af0edc-6eeb-11ea-925b-a0c3cdbe1130.shtml.
2. Driggin E, Madhavan MV, Bikdeli B, Chuich T, Laracy J, Bondi-Zoccai G, et al. Cardiovascular Considerations for Patients, Health Care Workers, and Health Systems During the Coronavirus Disease 2019 (COVID-19) Pandemic. J Am Coll Cardiol. 2020.
3. Zhou F, Yu T, Du R, Fan G, Liu Y, Liu Z, et al. Clinical course and risk factors for mortality of adult inpatients with COVID-19 in Wuhan, China: a retrospective cohort study. Lancet. 2020;395(10229):1054-62.
4. Tang N, Bai H, Chen X, Gong J, Li D, Sun Z. Anticoagulant treatment is associated with decreased mortality in severe coronavirus disease 2019 patients with coagulopathy. J Thromb Haemost. 2020.

5. Copin MC, Parmentier E, Duburcq T, Poissy J, Mathieu D, Lille C-I, et al. Time to consider histologic pattern of lung injury to treat critically ill patients with COVID-19 infection. Intensive Care Med. 2020.
6. Bikdeli B, Madhavan MV, Jimenez D, Chuich T, Dreyfus I, Driggin E, et al. COVID-19 and Thrombotic or Thromboembolic Disease: Implications for Prevention, Antithrombotic Therapy, and Follow-up. J Am Coll Cardiol. 2020.
7. Wynants L, Van Calster B, Bonten MMJ, Collins GS, Debray TPA, De Vos M, et al. Prediction models for diagnosis and prognosis of covid-19 infection: systematic review and critical appraisal. BMJ. 2020;369:m1328.
8. Chong PY, Chui P, Ling AE, Franks TJ, Tai DY, Leo YS, et al. Analysis of deaths during the severe acute respiratory syndrome (SARS) epidemic in Singapore: challenges in determining a SARS diagnosis. Arch Pathol Lab Med. 2004;128(2):195-204.
9. Madjid M, Safavi-Naeini P, Solomon SD, Vardeny O. Potential Effects of Coronaviruses on the Cardiovascular System: A Review. JAMA Cardiol. 2020.
10. Agarwal PP, Cinti S, Kazerooni EA. Chest radiographic and CT findings in novel swine-origin influenza A (H1N1) virus (S-OIV) infection. AJR Am J Roentgenol. 2009;193(6):1488-93.
11. Harms PW, Schmidt LA, Smith LB, Newton DW, Pletneva MA, Walters LL, et al. Autopsy findings in eight patients with fatal H1N1 influenza. Am J Clin Pathol. 2010;134(1):27-35.
12. Dimakakos E, Grapsa D, Vathiotis I, Papaspiliou A, Panagiotarakou M, Manolis E, et al. H1N1-Induced Venous Thromboembolic Events? Results of a Single-Institution Case Series. Open Forum Infect Dis. 2016;3(4):ofw214.
13. Danzi G, Loffi M, Galeazzi G, Gherbesi E. Acute Pulmonary Embolism and COVID-19 Pneumonia: A Random Association? Eur Heart J. 2020.
14. Lodigiani C, Iapichino G, Carenzo L, Cecconi M, Ferrazzi P, Sebastian T, et al. Venous and arterial thromboembolic complications in COVID-19 patients admitted to an academic hospital in Milan, Italy. Thromb Res. 2020.
15. Klok FA, Kruip M, van der Meer NJM, Arbous MS, Gommers D, Kant KM, et al. Incidence of thrombotic complications in critically ill ICU patients with COVID-19. Thromb Res. 2020.
16. Cui S, Chen S, Li X, Liu S, Wang F. Prevalence of venous thromboembolism in patients with severe novel coronavirus pneumonia. J Thromb Haemost. 2020.
17. Llitjos JF, Leclerc M, Chochois C, Monsallier JM, Ramakers M, Auvray M, et al. High incidence of venous thromboembolic events in anticoagulated severe COVID-19 patients. J Thromb Haemost. 2020.
18. Thomas W, Varley J, Johnston A. Thrombotic complications of patients admitted to intensive care with COVID19 at a teaching hospital in the United Kingdom. 2020.
19. Saba L, Sverzellati N. Is COVID Evolution Due to Occurrence of Pulmonary Vascular Thrombosis? J Thorac Imaging. 2020.
20. Klok FA. Incidence of thrombotic complications in critically ill ICU patients with COVID-19. Thromb Res.
21. Cui S. Prevalence of venous thromboembolism in patients with severe novel coronavirus pneumonia. J Thromb Haemost.
22. https://www.ilsecoloxix.it/basso-piemonte/2020/03/25/news/ad-alessandria-il-primo-intervento-su-paziente-intubato-per-covid-19-1.38635464.
23. https://www.ildolomiti.it/cronaca/2020/coronavirus-altri-17-decessi-in-trentino-morto-un-28enne-ha-avuto-una-embolia-polmonare.
24. https://www.thelocal.fr/20200306/coronavirus-what-do-we-know-so-far-about-the-people-who-have-died-in-france.
25. Senni M. COVID-19 experience in Bergamo, Italy. Eur Heart J. 2020.
26. Helms J, Tacquard C, Severac F. High risk of thrombosis in patients in severe SARS-CoV-2 infection: a multicenter prospective cohort study. Intensive Care Medicine. 2020.
27. Poissy J, Goutay J, Caplan M. Pulmonary Embolism in COVID-19 Patients: Awareness of an Increased Prevalence. Circulation. 2020.
28. Schmidt M, Horvath-Puho E, Thomsen RW, Smeeth L, Sorensen HT. Acute infections and venous thromboembolism. J Intern Med. 2012;271(6):608-18.
29. Schimanski S, Linnemann B, Luxembourg B, Seifried E, Jilg W, Lindhoff-Last E, et al. Cytomegalovirus infection is associated with venous thromboembolism of immunocompetent adults--a case-control study. Ann Hematol. 2012;91(4):597-604.
30. Avnon LS, Munteanu D, Smoliakov A, Jotkowitz A, Barski L. Thromboembolic events in patients with severe pandemic influenza A/H1N1. Eur J Intern Med. 2015;26(8):596-8.

31. Samama MM, Cohen AT, Darmon JY, Desjardins L, Eldor A, Janbon C, et al. A comparison of enoxaparin with placebo for the prevention of venous thromboembolism in acutely ill medical patients. Prophylaxis in Medical Patients with Enoxaparin Study Group. N Engl J Med. 1999;341(11):793-800.
32. Spyropoulos AC, Ageno W, Albers GW, Elliott CG, Halperin JL, Hiatt WR, et al. Rivaroxaban for Thromboprophylaxis after Hospitalization for Medical Illness. N Engl J Med. 2018;379(12):1118-27.
33. Rosenberg D, Eichorn A, Alarcon M, McCullagh L, McGinn T, Spyropoulos AC. External validation of the risk assessment model of the International Medical Prevention Registry on Venous Thromboembolism (IMPROVE) for medical patients in a tertiary health system. J Am Heart Assoc. 2014;3(6):e001152.
34. Madjid M, Miller CC, Zarubaev VV, Marinich IG, Kiselev OI, Lobzin YV, et al. Influenza epidemics and acute respiratory disease activity are associated with a surge in autopsy-confirmed coronary heart disease death: results from 8 years of autopsies in 34,892 subjects. Eur Heart J. 2007;28(10):1205-10.
35. Bonow RO, Fonarow GC, O'Gara PT, Yancy CW. Association of Coronavirus Disease 2019 (COVID-19) With Myocardial Injury and Mortality. JAMA Cardiol. 2020.
36. Wang D, Hu B, Hu C, Zhu F, Liu X, Zhang J, et al. Clinical Characteristics of 138 Hospitalized Patients With 2019 Novel Coronavirus-Infected Pneumonia in Wuhan, China. JAMA. 2020.
37. Guo T, Fan Y, Chen M, Wu X, Zhang L, He T, et al. Cardiovascular Implications of Fatal Outcomes of Patients With Coronavirus Disease 2019 (COVID-19). JAMA Cardiol. 2020.
38. Shi S, Qin M, Shen B, Cai Y, Liu T, Yang F, et al. Association of Cardiac Injury With Mortality in Hospitalized Patients With COVID-19 in Wuhan, China. JAMA Cardiol. 2020.
39. Patel AB, Verma A. COVID-19 and Angiotensin-Converting Enzyme Inhibitors and Angiotensin Receptor Blockers: What Is the Evidence? JAMA. 2020.
40. Varga Z, Flammer AJ, Steiger P, Haberecker M, Andermatt R, Zinkernagel AS, et al. Endothelial cell infection and endotheliitis in COVID-19. Lancet. 2020.
41. ISTH interim guidance on recognition and management of coagulopathy in COVID-19. In press. DOI: 10.1111/JTH.14810. 2020.
42. Thachil J. The versatile heparin in COVID-19. J Thromb Haemost. https://doi.org/10.1111/jth.14821.
43. Frizelle S, Schwarz J, Huber SA, Leslie K. Evaluation of the effects of low molecular weight heparin on inflammation and collagen deposition in chronic coxsackievirus B3-induced myocarditis in A/J mice. Am J Pathol. 1992;141(1):203-9.
44. Lang J, Yang N, Deng J, Liu K, Yang P, Zhang G, et al. Inhibition of SARS pseudovirus cell entry by lactoferrin binding to heparan sulfate proteoglycans. PLoS One. 2011;6(8):e23710.
45. Thrombosis-UK. Practical guidance for the prevention of thrombosis and management of coagulopathy and disseminated intravascular coagulation of patients infected with COVID-19. Available at: https://thrombosisuk.org/covid-19-thrombosis.php.
46. Connors JM, Levy JH. COVID-19 and its implications for thrombosis and anticoagulation. Blood. 2020.
47. van Wissen M, Keller TT, Ronkes B, Gerdes VE, Zaaijer HL, van Gorp EC, et al. Influenza infection and risk of acute pulmonary embolism. Thromb J. 2007;5:16.
48. Raskob G. Rivaroxaban for Extended Thromboprophylaxis After Hospitalization for Medical Illness: Pooled Analysis of Mortality and Major Thromboembolic Events in 16,496 Patients From the MAGELLAN and MARINER Trials. Available at: https://www.ahajournals.org/doi/10.1161/circ.140.suppl_1.12863.
49. Bajaj NS, Vaduganathan M, Qamar A, Gupta K, Gupta A, Golwala H, et al. Extended prophylaxis for venous thromboembolism after hospitalization for medical illness: A trial sequential and cumulative meta-analysis. PLoS Med. 2019;16(4):e1002797.
50. Cimminiello C, Prandoni P, Agnelli G, Di Minno G, Polo Friz H, Scaglione F, et al. Thromboprophylaxis with enoxaparin and direct oral anticoagulants in major orthopedic surgery and acutely ill medical patients: a meta-analysis. Intern Emerg Med. 2017;12(8):1291-305.
51. van Es N, Coppens M, Schulman S, Middeldorp S, Buller HR. Direct oral anticoagulants compared with vitamin K antagonists for acute venous thromboembolism: evidence from phase 3 trials. Blood. 2014;124(12):1968-75.
52. Posch F, Konigsbrugge O, Zielinski C, Pabinger I, Ay C. Treatment of venous thromboembolism in patients with cancer: A network meta-analysis comparing efficacy and safety of anticoagulants. Thromb Res. 2015;136(3):582-9.
53. Zheng J, Chen Q, Fu J, Lu Y, Han T, He P. Critical appraisal of international guidelines for the prevention and treatment of pregnancy-associated venous thromboembolism: a systematic review. BMC Cardiovasc Disord. 2019;19(1):199.

54. Anderson DR, Morgano GP, Bennett C, Dentali F, Francis CW, Garcia DA, et al. American Society of Hematology 2019 guidelines for management of venous thromboembolism: prevention of venous thromboembolism in surgical hospitalized patients. Blood Adv. 2019;3(23):3898-944.
55. Witt DM, Nieuwlaat R, Clark NP, Ansell J, Holbrook A, Skov J, et al. American Society of Hematology 2018 guidelines for management of venous thromboembolism: optimal management of anticoagulation therapy. Blood Adv. 2018;2(22):3257-91.
56. Bates SM, Rajasekhar A, Middeldorp S, McLintock C, Rodger MA, James AH, et al. American Society of Hematology 2018 guidelines for management of venous thromboembolism: venous thromboembolism in the context of pregnancy. Blood Adv. 2018;2(22):3317-59.
57. Schunemann HJ, Cushman M, Burnett AE, Kahn SR, Beyer-Westendorf J, Spencer FA, et al. American Society of Hematology 2018 guidelines for management of venous thromboembolism: prophylaxis for hospitalized and nonhospitalized medical patients. Blood Adv. 2018;2(22):3198-225.
58. Turpie AG. Thrombosis prophylaxis in the acutely ill medical patient: insights from the prophylaxis in MEDical patients with ENOXaparin (MEDENOX) trial. Am J Cardiol. 2000;86(12B):48M-52M.
59. Konstantinides SV, Meyer G, Becattini C, Bueno H, Geersing GJ, Harjola VP, et al. 2019 ESC Guidelines for the diagnosis and management of acute pulmonary embolism developed in collaboration with the European Respiratory Society (ERS): The Task Force for the diagnosis and management of acute pulmonary embolism of the European Society of Cardiology (ESC). Eur Respir J. 2019;54(3).
60. Eriksson BI, Borris LC, Friedman RJ, Haas S, Huisman MV, Kakkar AK, et al. Rivaroxaban versus enoxaparin for thromboprophylaxis after hip arthroplasty. N Engl J Med. 2008;358(26):2765-75.
61. Anderson DR, Dunbar M, Murnaghan J, Kahn SR, Gross P, Forsythe M, et al. Aspirin or Rivaroxaban for VTE Prophylaxis after Hip or Knee Arthroplasty. N Engl J Med. 2018;378(8):699-707.
62. Cohen AT, Spiro TE, Buller HR, Haskell L, Hu D, Hull R, et al. Rivaroxaban for thromboprophylaxis in acutely ill medical patients. N Engl J Med. 2013;368(6):513-23.
63. Prandoni P, Siragusa S, Girolami B, Fabris F, Group BI. The incidence of heparin-induced thrombocytopenia in medical patients treated with low-molecular-weight heparin: a prospective cohort study. Blood. 2005;106(9):3049-54.
64. Schulman S, Kearon C, Subcommittee on Control of Anticoagulation of the S, Standardization Committee of the International Society on T, Haemostasis. Definition of major bleeding in clinical investigations of antihemostatic medicinal products in non-surgical patients. J Thromb Haemost. 2005;3(4):692-4.
65. Kakkar AK, Cimminiello C, Goldhaber SZ, Parakh R, Wang C, Bergmann JF, et al. Low-molecular-weight heparin and mortality in acutely ill medical patients. N Engl J Med. 2011;365(26):2463-72.
66. Hull RD, Schellong SM, Tapson VF, Monreal M, Samama MM, Nicol P, et al. Extended-duration venous thromboembolism prophylaxis in acutely ill medical patients with recently reduced mobility: a randomized trial. Ann Intern Med. 2010;153(1):8-18.
